# The Dlx5 and Foxg1 transcription factors, linked via miRNA-9 and -200, are required for the development of the olfactory and GnRH system

**DOI:** 10.1016/j.mcn.2015.04.007

**Published:** 2015-09

**Authors:** Giulia Garaffo, Daniele Conte, Paolo Provero, Daniela Tomaiuolo, Zheng Luo, Patrizia Pinciroli, Clelia Peano, Ilaria D'Atri, Yorick Gitton, Talya Etzion, Yoav Gothilf, Dafne Gays, Massimo M. Santoro, Giorgio R. Merlo

**Affiliations:** a Dept. Molecular Biotechnology and Health Sciences, University of Torino, Italy; b Doctorate School in Molecular Medicine, Dept. Medical Biotechnology Translational Medicine (BIOMETRA), University of Milano, Italy; c Inst. of Biomedical Technology, National Research Council, ITB-CNR Segrate (MI) Italy; d UMR7221 CNRS/MNHN - Evolution des régulations endocriniennes - Paris, France; e Dept. Neurobiology, George S. Wise Faculty of Life Sciences, Tel-Aviv University, Tel-Aviv 69978, Israel; f VIB, Vesalius Research Center, KU Leuven, Belgium

**Keywords:** Olfactory development, Neuronal differentiation, microRNA, GnRH, Dlx, Foxg1, Kallmann syndrome

## Abstract

During neuronal development and maturation, microRNAs (miRs) play diverse functions ranging from early patterning, proliferation and commitment to differentiation, survival, homeostasis, activity and plasticity of more mature and adult neurons. The role of miRs in the differentiation of olfactory receptor neurons (ORNs) is emerging from the conditional inactivation of *Dicer* in immature ORN, and the depletion of all mature miRs in this system. Here, we identify specific miRs involved in olfactory development, by focusing on mice null for *Dlx5*, a homeogene essential for both ORN differentiation and axon guidance and connectivity. Analysis of miR expression in *Dlx5*^−/−^ olfactory epithelium pointed to reduced levels of *miR-9*, *miR-376a* and four *miRs* of the -*200* class in the absence of *Dlx5*. To functionally examine the role of these miRs, we depleted *miR-9* and *miR-200* class in reporter zebrafish embryos and observed delayed ORN differentiation, altered axonal trajectory/targeting, and altered genesis and position of olfactory-associated GnRH neurons, i.e. a phenotype known as Kallmann syndrome in humans. *miR-9* and *miR-200*-class negatively control *Foxg1* mRNA, a fork-head transcription factor essential for development of the olfactory epithelium and of the forebrain, known to maintain progenitors in a stem state. Increased levels of z-*foxg1* mRNA resulted in delayed ORN differentiation and altered axon trajectory, in zebrafish embryos. This work describes for the first time the role of specific miR (-9 and -200) in olfactory/GnRH development, and uncovers a Dlx5–Foxg1 regulation whose alteration affects receptor neuron differentiation, axonal targeting, GnRH neuron development, the hallmarks of the Kallmann syndrome.

## Highlights

• Dlx5 controls the expressions of *miR9* and *miR-200*, which target the *Foxg1* mRNA
• miR-9 and -200 are needed for olfactory neurons differentiation and axon extension
• miR-9 and -200 are required for the genesis and position of GnRH neurons.
• Altered expression of miR-9 and -200 might contribute to the Kallmann disease.

## 1 Introduction

During mammalian embryonic development, olfactory receptor neurons (ORNs) are specified and differentiated within two distinct neuroepithelial regions: the main olfactory epithelium (OE) and the Vomero-Nasal (VN) epithelium. Although similar, OE- and VN-derived mature neurons express distinct classes of odorant receptors and carry out different odour-transducing functions. Such molecular and physiological distinctions are apparently maintained in all vertebrates.

During their early differentiation, immature ORNs extend their axons to reach the anterior forebrain and contact projection neurons. In the mouse embryo, overt axonal extension begins around E9–E10, and is accompanied by pools of migratory cells including the GnRH neurons, which reach the olfactory bulbs (OB) around E12–E13 and subsequently reach their final destination in the medial preoptic area and other areas of the hypothalamus (Astic et al., 1998; Cariboni et al., 2007; Forni et al., 2011; Tarozzo et al., 1995; Whitlock et al., 2006; Wray et al., 1989). Defects in olfactory development and GnRH neuron migration are thought to be the primary cause of the congenital disorder known as Kallmann syndrome (KS); this notion is supported by various mutant mouse phenotypes (see below) and by the observation of a single human foetus affected by KS (Schwanzel-Fukuda and Pfaff, 2002). KS is characterized by central hypogonadotropic hypogonadism (CHH) combined with a varying degree of anosmia and other disturbancies. Several protein-coding genes are known to be mutated in KS and/or in normosmic CHH (nCHH) patients, including *KAL1*, *FGFR1*, *FGF8*, *PROK-2*, *PROKR-2*, *Kiss1R*/*GPR54*, *NELF*, *CHD7*, *GnRH-R*, *GnRH-R*, *HS6ST1*, *TAC3*, *TACR3*, *SOX10*, *SEMA3a* and 5 members of the “FGF8-synexpressome” (Bonomi et al., 2012; Cadman et al., 2007; Cariboni and Maggi, 2006; Dode and Hardelin, 2009; Hardelin and Dode, 2008; Hu et al., 2003; Miraoui et al., 2013; Semple and Topaloglu, 2010; Topaloglu and Kotan, 2010). However, mutations in these genes account for less than 40% of the cases. It is expected, therefore, that more KS and CHH disease genes remain to be identified. Likewise, several mutant mouse strains display a KS-like phenotype (Berghard et al., 2012; Cariboni et al., 2011; Corradi et al., 2003; Hanchate et al., 2012; Hardelin and Dode, 2008; Hirata et al., 2006; Ikeda et al., 2007; Laub et al., 2006; Levi et al., 2003; Long et al., 2003; Matsumoto et al., 2006; Merlo et al., 2007; Ng et al., 2005; Shimizu and Hibi, 2009; Watanabe et al., 2009; Yoshida et al., 1997), but these all represent loss-of-function mutations in protein-coding genes.

It is increasingly being recognized that biological processes are governed by complex regulatory modules and networks of molecular interactors, rather than simplistically by individual genes with individual functions. In these networks, non-coding RNAs (miR, lncRNAs, linc-RNAs, anti-sense RNAs and pseudogenes) play an important role (Arora et al., 2013; Esteller, 2011; Konopka, 2011; Mayanil, 2013; Ng et al., 2013; O'Brien et al., 2012; Salmena et al., 2011; Satoh, 2012; Schonrock et al., 2012). Thus, it is conceivable that mutations or misexpression of non-coding RNAs could participate in the molecular pathogenesis of KS/nCHH. Gaining knowledge on the RNA networks and regulations underlying olfactory differentiation, neuronal connectivity and guidance would be of great importance.

MicroRNAs (miRs) represent a class of short non-coding RNAs that act as negative post-translational regulators on longer coding and non-coding RNAs (Bartel, 2004). Annealing of complementary sequences enables miR to induce degradation or inhibit translation of target mRNAs (Plasterk, 2006). The neuronal functions of miR range from patterning and cell differentiation during embryonic development to physiology of more mature and adult neurons, including their survival, homeostasis, activity and plasticity (Agostini et al., 2011; Aranha et al., 2011; Bian and Sun, 2011; Brett et al., 2011; Fiore et al., 2011; Gao, 2010; Gaughwin et al., 2011; Li et al., 2011; Luikart et al., 2011; Olde Loohuis et al., 2012; Shi et al., 2010). More specifically, a role of miRs in the development of sensory neurons, including olfactory sensory neurons, is beginning to emerge. In *Drosophila*, *miR-7* has been implicated in the differentiation of photoreceptor cells via regulation of the EGF receptor signalling (Li and Carthew, 2005). In *Caenorhabditis elegans miR-273* and *lsy-6* have been shown to be required for asymmetric expression of taste receptors in chemosensory neurons (Chang et al., 2004; Johnston and Hobert, 2003). In *Danio rerio* (zebrafish) the *miR-200*-class is required for the proliferation, differentiation and survival of ORNs (Choi et al., 2008). In *Xenopus laevis miR-124* regulates changes in the sensitivity of retinal ganglion cells' growth cones to the guidance signal SEMA3A (Baudet et al., 2011), implicated in the pathogenesis of KS (Cariboni et al., 2011; Hanchate et al., 2012). In the mouse, the conditional disruption of *Dicer* in the developing olfactory system results in impaired ORN differentiation and reduced survival (Choi et al., 2008), indicating that mature miRs are required for these processes; however, without revealing their identity.

Since the activity of single miR is context- and time-specific, their functions should be examined within these contexts. With this in mind we generated high-throughput data from the developing olfactory system, focusing on the *Dlx5* homeogene: its targeted inactivation leads to a fully penetrant KS-related defects consisting in delayed ORN differentiation, impaired axonal connectivity and failure of GnRH neurons to reach the forebrain (Levi et al., 2003; Long et al., 2003; Merlo et al., 2007). We screened for miR expression in ORNs, comparing wild-type vs *Dlx5* mutant tissues, and identified *miR-9* and *miR 200*-class as the molecular link between Dlx5 and Foxg1. Using reporter zebrafish strains to visualize the embryonic olfactory axons (Miyasaka et al., 2005; Sato et al., 2005; Yoshida et al., 2002) or the GnRH + neurons (Abraham et al., 2008, 2009, 2010), we show that *miR-9* and *miR-200*-class play a role in ORN differentiation and axonal organization. We also show that these miRs are required for early GnRH neuron genesis and position. Thus we have identified a novel miR-based regulation that participates in the control of olfactory development, axon connectivity and GnRH neuron development.

## 2 Materials and method

### 2.1 Dlx5 mutant mouse strain

All procedures using mice were approved by the Ethical Committee of the University of Torino, and by the Italian Ministry of Health. Mice with targeted disruption of *Dlx5* have been previously reported (Acampora et al., 1999). The null allele, denominated *Dlx5^lacZ^*, allows for detection of the *Dlx5*-expressing cells by staining for β-galactosidase (β-gal) expression. The olfactory phenotype has been previously characterized (Levi et al., 2003; Long et al., 2003; Merlo et al., 2007). *Dlx5*^+/−^ (heterozygous) males and females were crossed, and generated the expected Mendelian ratios of embryos with genotype WT, *Dlx5*^+/−^ and *Dlx5*^−/−^. Pregnant females were sacrificed at the chosen embryonic age by cervical dislocation.

### 2.2 Collection of embryonic olfactory epithelia and RNA extraction

Embryos were collected clean of extra-embryonic tissues (used for genotyping) by manual dissection, transferred in RNAse-free PBS, and further dissected to separate the head. This was then included in 3% low-melting agarose (Sigma-Aldrich) in PBS, let harden and vibratome-sectioned at 250 μm thickness. Sections were transferred in RNase-free PBS, and manually dissected with fine pins to collect the OE or the VNO epithelia. For the *Dlx5* mutant tissues, the entire epithelial lining of the nasal cavity was collected, since it is not possible to discriminate the olfactory vs. the respiratory epithelium. The excised tissues were individually collected in RNA-later (Life Technologies AM7020) and stored at -20 until extraction. The collected tissues with the same genotype were pooled into three samples, used to extract total RNA with the TRIzol reagent (Life Technologies), following the manufacturer's instructions. Correct pooling was further verified by RT-PCR for the *Dlx5* mRNA. RNA samples were quantified using a NanoDrop1000 spectrophotometer (Nanodrop Technologies, Inc.), and analysed by capillary electrophoresis on an Agilent Bioanalyzer. Only samples showing an RNA Integrity Number > 5 were further processed. The quality of small RNAs was further assayed by using a small RNA Chip Bioanalyzer (Agilent).

### 2.3 miR profiling and data analysis

RNA samples were labelled using the one-colour method, with the miRNA Complete Labelling and Hybridization kit (Agilent). For the profiling, the mouse 8x15K arrays were used (Agilent) on a mouse V2 microarray platform. These arrays comprise probes for 627 mature mouse miRs and 39 viral miRs. Data were extracted using conventional spot-recognition and significantly-above-background tools; the signal intensity was normalized across samples using the Lowess cyclic normalization algorithm. Differentially expressed miRs (DEM) were detected using the SAM two-class unpaired statistical tool, using a FDR = 5%. Of the 627 mouse miR probes present on the arrays, 118 miRNA were found to be expressed significantly above the background.

### 2.4 Softwares and databases used

For preliminary Gene Ontology (GO) analyses we used DAVID (http://david.abcc.ncifcrf.gov/) and KEGG (http://www.genome.jp/kegg/pathway.html). For improved categorization and visualization, we used ClueGO (Bindea et al., 2009). To examine the embryonic expression of individual RefSeq we used the publicly available transcriptome-wide in situ expression databases Genepaint (www.genepaint.org) and Eurexpress (www.eurexpress.org). For the prediction of miR targets we used TargetScan6.2 (www.targetscan.org) and Hoctar, a miR target prediction tool based on a combination of miR-mRNA co-expression and seed sequence recognition (Gennarino et al., 2011). For photo-documentation, digital micrographic images were contrast balanced and colour matched using PhotoShop 7 (Adobe), cropped, rotated and assembled into figures with QuarkXPress (Pantone).

### 2.5 Additional softwares and databases

Ensembl Genome Browser, http://www.ensembl.org/index.html

UCSC Genome Browser, http://genome.ucsc.edu

RefSeq, http://www.ncbi.nlm.nih.gov/RedSeq

Mouse Genome Informatix, http://www.informatics.jax.org/

On-line Mendelian Inheritance in Man (OMIM), http://www.omim.org/

TS-CoExp Browser, *http://www.mbcunito.it/cbu/ts-coexp*.

### 2.6 Molecular validation of the DEM

To verify the up- and down-modulations of selected miRs, additional embryonic olfactory epithelia from WT and *Dlx5*^−/−^ embryos (age E12.5) were collected in RNA-later, genotyped and pooled as above. Relative abundance of selected miR was determined using the single-miR assay RT-qPCR assays (TaqMan miR assays, Life-Technologies), with the AB7900 equipment (Applied Biosystem). Specifically, the following assays were employed: miR-9: 000583, miR-200a: 000502, miR-200b: 002251, miR-141: 000463, miR-429: 001077, miR-376a: 001069, miR-130b: 002460, miR-450a/5p: 002303 and U6: 001973. Experiments were done with technical triplicates, repeated on two independent biological samples; snU6 was used as endogenous control and for normalization. Relative miR abundance was calculated with the ΔΔCt formula.

### 2.7 Real-Time qPCR analysis for coding mRNAs

Relative mRNA abundance was determined by Real-Time qPCR. Total RNA (500 ng) was reverse-transcribed at 42 °C with Reverse Transcriptase (Promega), amplified with the GoTaq qPCR Master Mix (Promega), and detected with the Universal Primer System (UPS, Roche) on the AB7900 equipment (Applied Biosystem). The abundance of *TATA-binding protein* (*TBP*) and of *GAPDH* mRNAs were used for normalization. Primers were designed with the dedicated UPS on-line tool (Roche) and provided in Suppl. Table I. Experiments were repeated twice on independent samples, every point was done in triplicate. Data analysis was performed using the ΔΔCt formula, with ABI software, version 2.1 (Applied Biosystems).

### 2.8 Identification of Dlx5 binding sites and chromatin immunoprecipitation

Dlx5 binding sites and putative target genes were predicted essentially as described (Vieux-Rochas et al., 2013). Briefly, the PWM provided by JASPAR (PH0024.1) was used to score sites using standard log-likelihood ratios. Only those sites conserved in at least two vertebrate species were considered. For chromatin immunoprecipitation (ChIP) we used the human SHSY-5Y neuroblastoma cells, which express low endogenous levels of *Dlx5*, *miR-9* and *miR-200*, transfected with 5 μg of *DLX5-myc*-tag expression vector (from Open-Biosystem) or with the same vector in which the Q178P mutation (Shamseldin et al., 2012) was introduced (BioFab, Rome, sequence verified). Chromatin was crosslinked, sonicated, precipitated with anti-myc TAG antibody (SantaCruz, sc-40) and de-crosslinked according to instructions (EZ Magna ChipG, Millipore). Genomic fragments comprising the predicted Dlx5 putative binding sites were PCR-amplified with primer flanking the site (sequences in Suppl. Table II). Chromatin from cells transfected with an empty vector, or with a vector expressing the Q178P *DLX5*, was used for comparison. ChIP with an irrelevant IgG was also used as negative control. Total chromatin was used as positive control (input).

### 2.9 Transfection of pre-miRs and anti-miRs

To downmodulate endogenously expressed *miR-9* and *miR-200* we used the commercially available Ambion anti-miR inhibitors (Life Technologies). To overexpress *miR-9* and *miR-200* exogenously we used commercially available Ambion pre-miR precursors (Life Technologies). The human neuroblastoma cells SH-SY5Y were plated in 6-well plates at 50% confluency and 24 h later were transfected with 100 nM of the anti-miR or 75 nM of the pre-miR vector, using the Lipofectamine 2000 reagent (Life Technologies), according to the manufacturer's instructions. Cells were examined for miR over-expression or knock-down 48 hrs after transfection, by single-assay miR Real-Time qPCR (Applied Biosystem).

### 2.10 Antibodies and immunoblotting

For Western immunoblotting, the following primary antibodies were used: anti-Foxg1 (1:1000, Abcam) and anti-vinculin. Secondary antibody was horseradish peroxydase-conjugated anti-mouse and anti-rabbit (Sigma, 1:250). Total proteins were extracted either from transfected SH-SY5Y cells (following trypsinization and centrifugation) or from the forebrains of E16.5 embryos (following dissection and quickly freezing in liquid N_2_) using the same procedure. Essentially, cells were lysed with an electrical tissue homogenizer (Turax) in modified RIPA buffer composed as follows: 150 mM NaCl, 50 mM Tris, pH 7.5 with 1% Triton X-100, 0.5% Na deoxycholate, 0.1% SDS, 2 mM EDTA, 1 mM sodium orthovanadate, 1 mM sodium fluoride and protease inhibitors. Homogenates were centrifuged at 13,000 rpm for 10 min at 4 °C, extracts were quantified with the Bio-Rad Assay, then separated in 10% acrylamide gel and transferred to a PVDF membrane (Millipore). Membranes were saturated with BSA 5% in TBS-Tween-20 0.3%, washed in TBS-Tween, incubated with the primary antibodies ON at 4 °C, then washed in TBS-Tween, incubated with HRP-conjugated secondary antibodies (diluted 1:5000), washed and developed with chemo-luminescence reagent Luminata TM Forte Western HRP Substrate (Millipore). Images were acquired with the ChemiDoc system (Bio-Rad), exported and analysed with Quantity One software. Signals were normalized against vinculin.

### 2.11 miR in situ hybridization in tissue sections

WT and *Dlx5*^−/−^ mouse embryos were collected at 13.5 days post-coitum (E13.5), fixed in 4% PFA/0.1 M phosphate buffer (PB, pH 7.4) for 12–16 hrs, washed in PBS, dehydratated in methanol, processed for paraffin embedding and sectioned at 6 μm. Hybridization was carried out with DIG-labelled riboprobes that specifically detect the mature form of mouse *miR-9* and *miR-141* (Exiqon) in according with manufactory instruction. The sections were hybridized with the probe for 16 hrs, washed, incubated with an anti-DIG-AP antibody (Roche) and developed with NBT-BCIP (Sigma). To control the efficiency of the procedure and RNA preservation, we adjacent sections were hybridized with a probe that detects the *snU6* small ncRNA.

### 2.12 Zebrafish strains and treatments

All procedures using zebrafishes are authorized by the Ethical Committee of the University of Torino and the Italian Ministry of Health. The following two strains were used for visualization of the olfactory axons: *OMP^2k^:gap-CFP^rw034^* and *TRPC2^4.5k^:gap-Venus^rw037^* (Miyasaka et al., 2005; Sato et al., 2005; Yoshida et al., 2002). These strains were obtained from Drs. Nobuhiko Miyasaka and Yoshihiro Yoshihara (RIKEN Brain Science Institute, Japan). The *OMP::CFP* transgene labels the OE-type neurons of the fish olfactory placode, while the *Trpc2::Venus* transgene labels the VNO-type neurons (Sato et al., 2005). The fish strain *GnRH3::GFP* (Abraham et al., 2008, 2009, 2010) was obtained from Prof. Y. Zohar (Univ. Maryland Biotechnology Institute, Baltimore, USA).

Adult fishes were maintained, bred and genotyped according to standard procedures, kept under a 14 hrs-light and 10 hrs-dark photoperiod at approximately 28 °C. Allelic transmission followed the expected Mendelian ratios. Following fertilization, eggs were collected and embryos (WT or micro-injected) were grown in the presence of 0.003% 1-phenyl-2-thiourea (PTU) to prevent formation of melanin pigment. To down-modulate specific miRs we utilized a conventional antisense morpholino oligo (MO)-mediated strategy (from GeneTools, LLC, Philomat OR, USA) (Flynt et al., 2007; Kloosterman and Plasterk, 2006). For *z-dlx5a* two MOs were designed: one that prevented exon1–intron1 splicing, and consequently led a premature Stop codon upstream of the homeodomain, and a second one annealing on the ATG start codon and preventing translation. Anti-*z-miR-9* MO was designed with the on-line dedicated tool https://oligodesign.gene-tools.com/request/, while the anti-*z-miR-200* MO mix was as previously published (Choi et al., 2008). Sequences are provided in Suppl. Table III. The complete zebrafish *foxg1a* cDNA (gene ID 30274), including the CDS, the 5′ and the 3′ UTRs, was cloned in the pCS2 + plasmid, and the mRNA was in vitro transcribed, polyadenylated and capped with the mMESSAGE mMACHINE SP6 kit (Life Technologies).

Fertilized eggs were collected at one cell stage and injected under stereological examination with 4 ng of MO, in the presence of Phenol Red for further selection of the injected embryos. 48–72 h post fertilization (hpf) embryos were fixed with 4% PFA at 4 °C ON, washed in PBS and embedded in 4% low melting agarose, 0.1% Tween-20. The apical portion of the head was manually dissected from the rest of the embryo. The OMP::CFP + and the Trpc2::Venus + (YFP +) axons were viewed in a frontal plane. Confocal microscopy analysis was performed using Leica TCS SP5 (Leica Microsystems). Images were acquired as Z-stacks of 1 μm-thick optical sections. Digital micrograph images were contrast balanced and colour matched using PhotoShop 7 (Adobe), cropped, rotated and assembled into figures with QuarkXPress (Pantone).

GnRH3::GFP + neurons were examined on fixed 72 hpf embryos, at 20 × and 40 × magnification, by confocal microscopy. For counting, we followed the procedure detailed in Y. Zhao et al. (2013). Briefly, stacks of Z-slices at 40 × were used to manually count the GFP + neurons with the assistance of ImageJ image-processing software (from NIH), after adjustment of contrast and brightness. To avoid double-counting of the same neuron, each optical slice (1 μm-thick) was also individually reexamined. Merged images of all Z-slices confirmed the estimated number.

For time-lapse video recording, embryos were collected at 34 and 60 hpf, anaesthetized with tricain (standard dose) embedded in 0.8% low-melting agarose and examined by confocal microscopy using the following parameters: 20 × magnification, 10 Z-slices (5 µm each), acquisition every 20 min. for a total of 16 hrs. Laser intensity was adjusted to avoid photo-damage. Row images were adjusted for contrast and brightness, oriented and registered with ImageJ, videos were mounted with the same software.

## 3 Results

### 3.1 Differentially expressed miRs in Dlx5^−/−^ OE

**Fig. 1:**
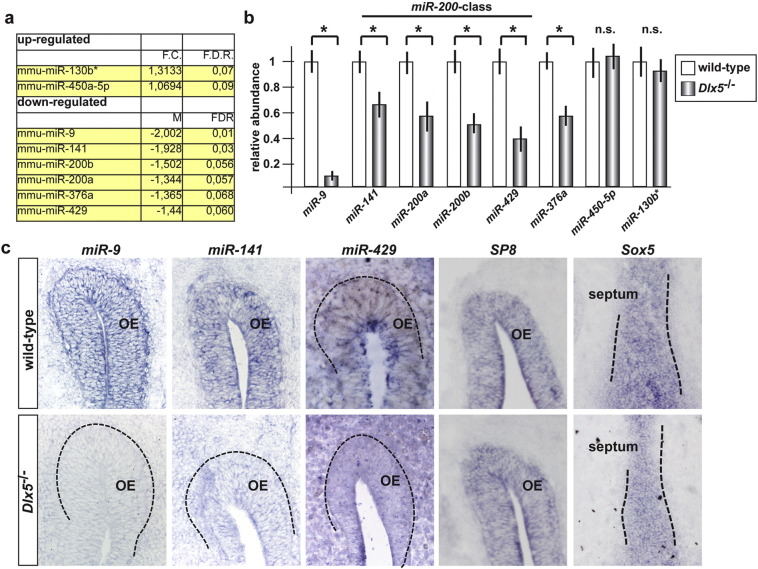
Differential miR expression in the *Dlx5*^−/−^ olfactory epithelium. a. Differentially expressed miRs (up- and down-regulated) in the OE of *Dlx5*^−/−^ embryos. The fold-change (F.C.) and the false-discovery rate (F.D.R.) are reported. b. Real-Time qPCR to quantify the abundance of individual miRs in the *Dlx5* mutant (solid bars) vs. WT (open bars) OE, to verify changes in abundance of those miRs found to be differentially expressed by array hybridization. For this experiment, newly collected samples were used. miRs of the *200-*class are indicated on top. Asterisks indicate statistical significance. n.s., not significant. c. in situ hybridization on coronal sections of WT (top panels) and *Dlx5*^−/−^ (bottom panels) embryos, at the age E12.5, to detect endogenous *miR-9*, *miR-141* and *miR-429*. Hybridization with probes detecting *SP8* (expressed in the OE) or *Sox5* (expressed in chondrogenic condensations) was carried out as control for RNA preservation (panels on the right). The olfactory epithelium (OE) and the septum are indicated (dotted lines). Note the reduced expression of all the tested *miRs* in the mutant specimens.

RNA samples from WT and *Dlx5*^−/−^ OE (age E12.5) were used to hybridize on Agilent arrays for the detection of mature mouse miRs. Of the 627 miR probes present on the arrays, 118 miRs were found to be expressed significantly above the background. We compared normal vs. *Dlx5*^−/−^ OE using both SAM two-class unpaired and the Rank Product statistical analyses, with a FDR = 5 %. We found eight miRs differentially expressed, six down-regulated (*miR-9*, *miR-141*, *miR-200a*, *miR-200b*, *miR-429* and *miR-376a*) and two up-regulated (*miR-450a-5p* and miR130b*) in the *Dlx5*^−/−^ OE (Fig. 1a). The most altered miR was *miR-9*, with a fold change of -2, while the other miRs showed a fold-change between − 1.9 and + 1.3.

To confirm the modulation of these miRs in the absence of *Dlx5*, we quantified their expression by Real-Time qPCR, using newly collected RNA samples from WT and *Dlx5*^−/−^ embryonic OE. The results confirmed a reduced level of all the down-regulated miRs, while did not confirm the up-regulated ones (Fig. 1b); hence, these were not further considered. As a further confirmation, we carried out in situ hybridization on sections of WT and *Dlx5*^−/−^ embryonic OE, at the age E12.5, to detect *miR-9*, *miR-141* and *miR-429*, using specific mouse DIG-labelled probes. We observed a reduction of *miR-9*, *miR-141* and *miR-429* signal in the *Dlx5*^−/−^ OE, compared to the WT (Fig. 1c), while hybridization with two positive controls, *Sp8* (expressed in the OE) and *Sox5* (expressed in chondrogenic condensations), yielded an equivalent positive signal in both genotypes, indicating adequate RNA preservation. *miR200a* and *miR200b* could not be tested by in situ hybridization due to high sequence conservation between all members of the *miR-200*-class.

### 3.2 Genomic regulation of miR-9 and miR-200 by Dlx5

**Fig. 2:**
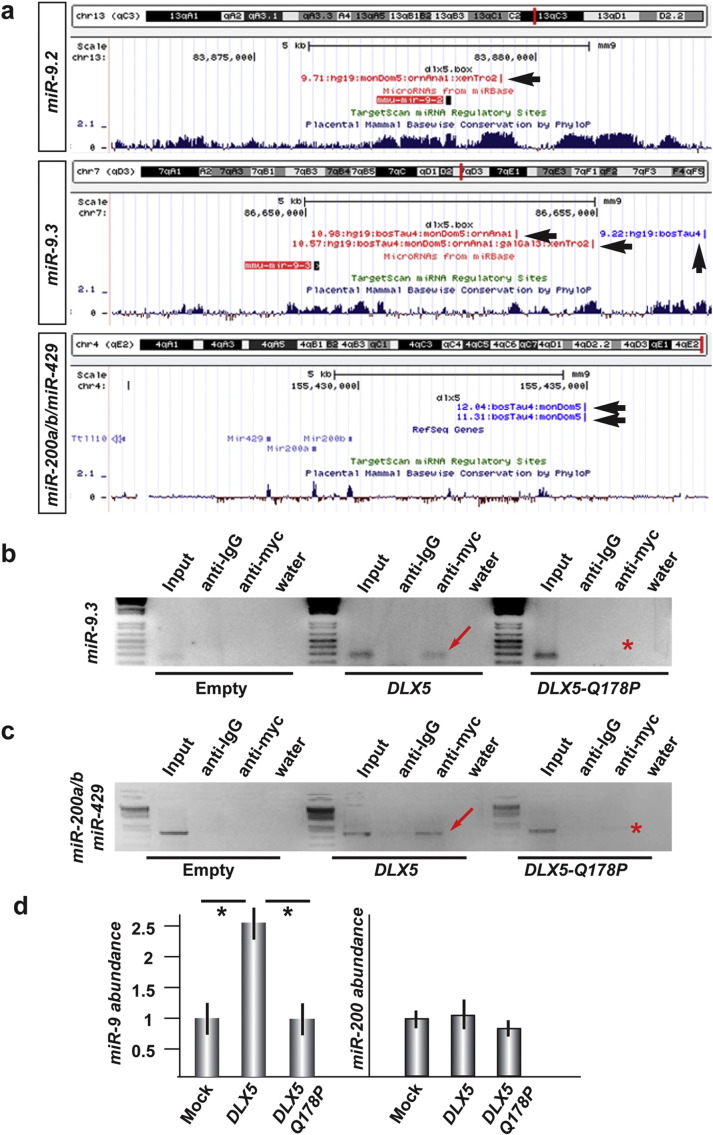
Dlx5 regulates the expression of *miR-9* and *miR-200-*class loci. a. Position of predicted Dlx5 binding sites near the *miR-9.2*, the *miR-9.3* and the *miR-200a*/*b*/*miR-429* loci (black arrowheads), annotated on the UCSC mouse genome browser. The position of the miR loci and the mammalian conservation are reported. Note that the predicted Dlx5 sites fall in conserved regions. b, c. ChIP analyses with anti-myc TAG antibody, of human genomic fragments encompassing the Dlx5 sites predicted for *miR-9.3* and *miR-200a*/*b*/*miR-429* (shown in a), upon transfection of human SH-SY5Y cells with an empty vector (left lanes), with a *DLX5-myc* (middle lanes) or with a Q178P mutant *DLX5-myc* (right lanes) expression vector. Enrichment is observed only when wild-type DLX5 (red arrows), and not Q178P mutant DLX5 (red asterisks), is transfected (red arrows). d. Quantitative determination of endogenous level of *miR-9* and *miR-200* in SH-SY5Y cells, upon exogenous expression of *DLX5-myc* or Q178P *DLX5-myc*. Expression of WT DLX5, but not Q178P mutant DLX5 results in increased levels of *miR-9*, but not *miR-200*.

The three loci *miR-9.1*, *-9.2* and -*9.3*, located on chromosomes 3, 13 and 17 respectively, generate identical mature miR when transcribed, referred to as “*miR-9*”. The *miR-200a*, -*200b* and -*429* loci are closely located on chromosome 4, while *miR-141* and *-200c* are closely located on chromosome 6. *miR-376a* is clustered with 16 other miRs on chromosome 12. Suppl. Fig. 1 provides a summary of the sequence and chromosomal location of the mentioned miRs. We sought to establish if any of the downmodulated miRs might be a direct Dlx5 transcriptional target. First we searched for the presence of Dlx5 binding sites near the identified miRs loci in genomic regions conserved between human, mouse and other mammalian species. We used a previously generated genome-wide prediction of Dlx5 binding sites, based on its Position-Weight Matrix present in the Jaspar database (Portales-Casamar et al., 2010; Vieux-Rochas et al., 2013). We predicted one Dlx5 binding site near the *miR-9.2* locus, located about 1.5 kb downstream, three sites near the *miR-9.3* locus, located about 4, 5 and 6 kb downstream, and two sites near the *miR-200a–200b-429* locus, located about 5 kb upstream (Fig. 2a). No Dlx5 binding site was predicted within a 50 kb range from the *miR-9.1*, *miR-141*, *miR-200c* and *miR-376a* loci.

To test whether the *DLX5* protein physically occupies the Dlx5 sites near the *miR-9.3* and *miR-200a*/*b*/*miR-429* loci, Chromatin Immuno-Precipitation (ChIP) analysis on these sites was performed. myc-tagged version of either the WT or the Q178P mutant *DLX5* were expressed in the SH-SY5Y human neuroblastoma cells, which express *DLX5*, *miR-9* and *miR-200* endogenously. The Q178P mutation falls in the DNA-binding domain of the protein, and has been found to co-segregate with ectrodactyly in a human family (Shamseldin et al., 2012). For both locations, an enrichment in the myc-immunoprecipitated chromatin in cells transfected with WT *DLX5* was observed, compared to cells transfected with the empty vector, while the transfection with *DLX5*-Q178P failed to show binding (Fig. 2b, c). The identity of the amplified ChIP fragments was sequence-verified.

To determine whether the forced expression of DLX5 may result in an upregulation of *miR-9* and *miR-200*-class RNAs, SH-SY5Y cells were transfected with myc-tagged wild-type *DLX5* or Q178P mutant *DLX5* expression vectors, and the relative abundance of *miR-9* and *miR-200* was quantified by Real-Time qPCR. The over-expression of *DLX5* induced a 2.5–3 fold increase in the abundance of *miR-9* in this system, while the Q178P mutant *DLX5* did not (Fig. 2d). On the contrary, *DLX5* overexpression did not induce changes in *miR-200* expression, either in SH-SY5Y (Fig. 2d) or in GN11 (neuroendocrine) or in U2OS (osteosarcoma) cells (data not shown). Thus, Dlx5 is likely to regulate the expression of *miR-9.3* directly, and the expression of *miR-200a*/*b*/*miR-429* indirectly. Alternatively the expression of *miR-200a*/*b*/*miR-429* could require additional transcription (co)factors not present in these cells.

### 3.3 Searching for functionally relevant targets of miR-9 and miR-200 clsss in the OE

*miR-9* is widely expressed in the forebrain and olfactory sensory system of the mouse embryo and has been implicated in neural development (La Torre et al., 2013; Shibata et al., 2011; C. Zhao et al., 2013). The miRs of the -*200* class are also expressed in the developing OE and are known to play a role in ORN differentiation (Choi et al., 2008). Since *miR-200a*, *miR-200b*, *miR-141* and *miR-429* share very similar seed sequences (Suppl. Fig. 1), they are predicted to bind to, and negatively regulate, common sets of RNA targets.

To compile lists of best predictable targets, we initially used TargetScan-6.2, a tool that uses sequence base-pairing and conservation criteria. We integrated the results obtained with TargetScan with Hoctar (Gennarino et al., 2011), a prediction tool that combines sequence base-pairing with co-expression and anti-correlated co-regulation criteria. With these two tools, we predicted the most reliable *miR-9* targets, and functionally classified the top scoring ones, to search for significantly enriched categories. The results were organized as “Biological Process”, “Molecular Function” and “Cellular Component” using the GeneOntology (GO)-based tool ClueGO (Bindea et al., 2009). For *miR-9* we detected only three enriched categories: regulation of cell differentiation, cell junction assembly and neuron development (Suppl. Tables IV A, B, C).

We carried out the same prediction and categorization analyses for the *miR* of the *-200* class; in this case the two subfamilies (*miR-141*/*200a* and *miR-200b*/*c*/*429*/*548a*) were examined separately, and indeed yielded lists which were similar but not identical. For *miR-141*/*200a*, the enriched categories included regulation of cell differentiation, neurogenesis, development and regulation of transcription (Suppl. Table VA, B, C). For *miR-200b*/*c*/*429*/*548a*, the enriched functional categories included forebrain generation of neuron, cell motility, projection development, regulation of locomotion, regulation of phosphorylation and regulation of transcription (Suppl. Table VIA, B, C).

We then searched for predicted targets that have seed sequences for two or three of the miRs downregulated in the absence of Dlx5. The analysis yielded, respectively, 54 and 30 mRNAs, whose functional annotation indicated an enrichment of the following categories: cell differentiation, neurogenesis, neuron differentiation, projection development and regulation of transcription (Suppl. Tables VIIA, B, C and VIIIA, B). Amongst these we noted *Foxp1* and *Foxg1*, which however were not differentially expressed in the *Dlx5^−/−^* OE with the adopted cut-off value and statistical parameters (Garaffo et al., 2013). Two possible explanations: either changes in the abundance of *miR-9* and *miR-200*-class cause changes in the abundance of target RNAs that are too modest to pass the imposed cut-off value, or these miRs preferentially affect translation and not stability of the target mRNAs.

Next we intersected the predicted *miR-9* and *miR-200*-class targets with the coding mRNAs found to be differentially expressed in the *Dlx5*^−/−^ OE compared to the WT (Garaffo et al., 2013). A significant enrichment of *miR-9* and *miR-200*-class target sequences was detected in the 3′ UTR of genes up-regulated in the *Dlx5*^−/−^ OE (Table 1A, B). Some of the *miR-9* and *miR-200*-class targets upregulated in the mutant OE (*Qk, Foxf2*) are mesenchymally-expressed rather than OE-expressed, while other targets were actually downregulated in the absence of *Dlx5* (*Akap6*, *Elmod1*, *Snap25*) (Table 1C). The overall poor anti-correlation between DEMs and DEGs may be explained considering that changes in the miR abundance not necessarily cause changes in the abundance of the target mRNA. These genes were not further considered.

### 3.4 miR-9 and miR-200-class regulate Foxg1

**Fig. 3:**
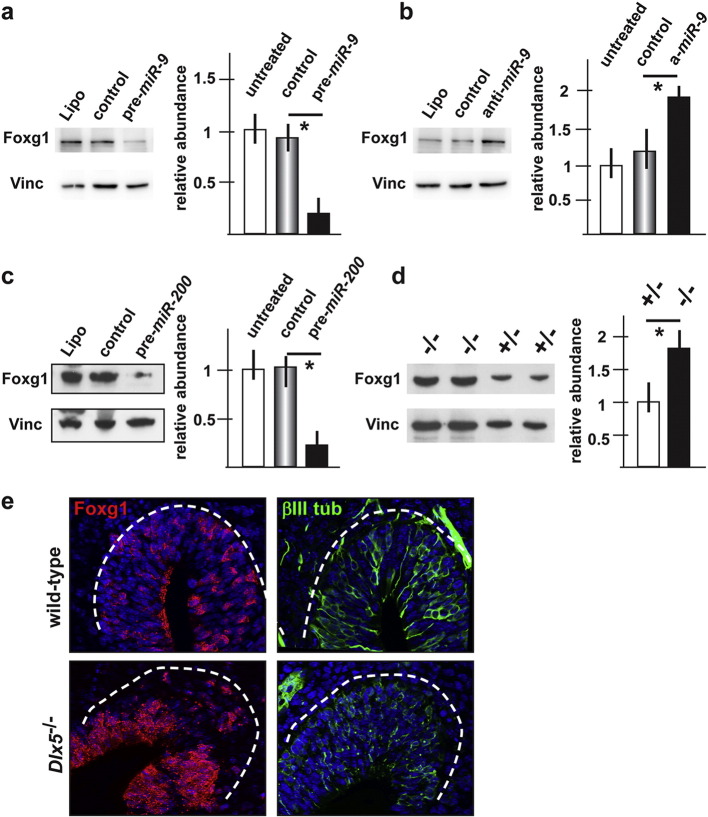
Foxg1 protein level is modulated by *miR-9* and *miR-200* class. a, b. Western blot analyses of endogenous Foxg1 protein level in SH-SY5Y cells, upon transfection of a pre-*miR-9* precursor (pre-miR-9) (a) or treatment of the cells with and anti-*miR-9* (b). Quantification was done using vinculin as house-keeping control, and is shown on the right of each blot. A down-modulation and an up-modulation of Foxg1 is observed, respectively, with the pre-miR and with the anti-miR. c. Western blot analyses of endogenous Foxg1 protein level in SH-SY5Y cells, upon transfection of a pre-*miR-200* vector. Relative quantification is shown on the right. A down-modulation of Foxg1 is observed. d. Western blot analyses of Foxg1 protein level in extracts of the forebrain of WT, *Dlx5*+/− and *Dlx5*^−/−^ embryos (E15.5), normalized against signal for vinculin. Relative quantification is shown on the right. Foxg1 protein level is increased 1.5-folds and 2.5-folds, respectively, in the heterozygous and the homozygous *Dlx5* mutant samples. e. Immunofluorescent staining of coronal sections of WT (top panels) or *Dlx5*^−/−^ (bottom panels) embryos, at the age E13.5, with anti-Foxg1 antibody (left panels, red staining) and anti-βIII-tubulin (right panels, green fluorescence) antibodies. The Foxg1 signal is increased n the OE of the mutant specimen, compared to the WT control.

The 3′ UTR of the mammalian and fish *Foxg1* mRNA contains seed sequences for *miR-9* and *miR-200* (Suppl. Fig. 2), and *Foxg1* mRNA has been proposed as a valid target of *miR-9* (Shibata et al., 2008). To determine whether *miR-9* and *miR-200*-class may modulate Foxg1 protein level, the effect of introduction of *pre-miR-9* or depletion of endogenous *miR-9* on Foxg1 protein level was assayed by Western blot analysis in SH-SY5Y cells, which express *DLX5*, *miR-9*, *miR-200*-class and *Foxg1* endogenously. These cells appear relatively more similar to the olfactory neurons (from a molecular point of view, and limited to these target genes) than other cellular models that we considered. The expression of pre*-miR-9* induced a 6-fold reduction in Foxg1 protein level, while expression of anti*-miR-9* induced a 2-fold increase in Foxg1 level (Fig. 3a,b). We also determined the level of endogenous *Foxg1* mRNA, by Real-Time qPCR, upon expression of pre*-miR-9* or anti-*miR-9*, and observed, respectively, a 2-fold decrease and a 2.5-fold increase in the relative *Foxg1* mRNA abundance (data not shown). In the same cells, the expression of pre*-miR-200* led to a 3.9-fold decrease in Foxg1 proteins level (Fig. 3c). Thus, both *miR-9* and *miR-200* negatively regulate Foxg1 protein level.

Next, the level of Foxg1 protein in extracts from the forebrain of *Dlx5*^+/−^ vs. *Dlx5*^−/−^ embryos, at the age E13.5, were compared by Western blot analysis. In the *Dlx5*^−/−^ samples we detected a 1.9-fold increase of Foxg1 protein, relative to the *Dlx5*^+/−^ sample (Fig. 3d). Finally, we examined the expression of Foxg1 protein on coronal sections of the nasal region of WT and *Dlx5*^−/−^ embryos, at the age E13.5, by immunostaining with anti-Foxg1 antibody. We observed an increase in Foxg1 signal in the *Dlx5*^−/−^ OE, compared to the WT (Fig. 3e). To confirm that Foxg1 staining corresponds to the OE in both genotypes, and to verify the delayed differentiation in the absence of Dlx5, we stained adjacent sections with the anti-βIII-tubulin antibody TuJ1. We detected a reduced βIII-tubulin staining in the *Dlx5*^−/−^ sections compared to the WT specimen. Thus, the level of Foxg1 protein is increased in the absence of Dlx5.

In summary, since *miR-9* and *miR-200-*class are down-modulated in the absence of Dlx5, while Foxg1 protein level is up-regulated, and since the 3′ UTR of the *Foxg1* mRNA is a predicted target of these miRs, we can infer that the Dlx5-miR-Foxg1 regulation is most likely a direct one.

### 3.5 Dlx5 is required for normal olfactory development in zebrafish embryos

Olfactory development and GnRH ontogenesis are highly conserved amongst vertebrates including zebrafish (Lohr and Hammerschmidt, 2011; Roa, 2013; Whitlock et al., 2006; Wierman et al., 2011). *D. rerio* (zebrafish) embryos are increasingly being used in developmental genetics, thus we opted to gain functional in vivo knowledge on the identified miRs, by using two lines of transgenic fishes that allow a direct visualization of the olfactory axons (Miyasaka et al., 2005; Yoshida et al., 2002). In these lines, two distinct fluorescent reporters are expressed in the two main populations of peripheral ORNs, and the reporter proteins are efficiently transported in their axons (Suppl. Fig. 3). In one strain *CFP* is expressed under the control of *OMP* promoter, and marks the majority of ORNs lying in the basal portion of the embryonic OE, projecting their axons to the dorsal OB. In the other strain, the *Venus* (*YFP*) reporter is expressed under the control of the *Trpc2* promoter and marks a sub-population of sensory neurons that occupies the apical part of the OE and projects to the ventro-lateral OB. The fish CFP + and the YFP + neurons are thought to correspond, respectively, to the OE and VNO receptors in the mammalian system (Sato et al., 2005); however, in the fish embryos these two sensory neuron populations originate from the olfactory placode but then remain intermingled and occupy the same neuroepithelium, while in the mouse they occupy distinct nasal structures.

**Fig. 4:**
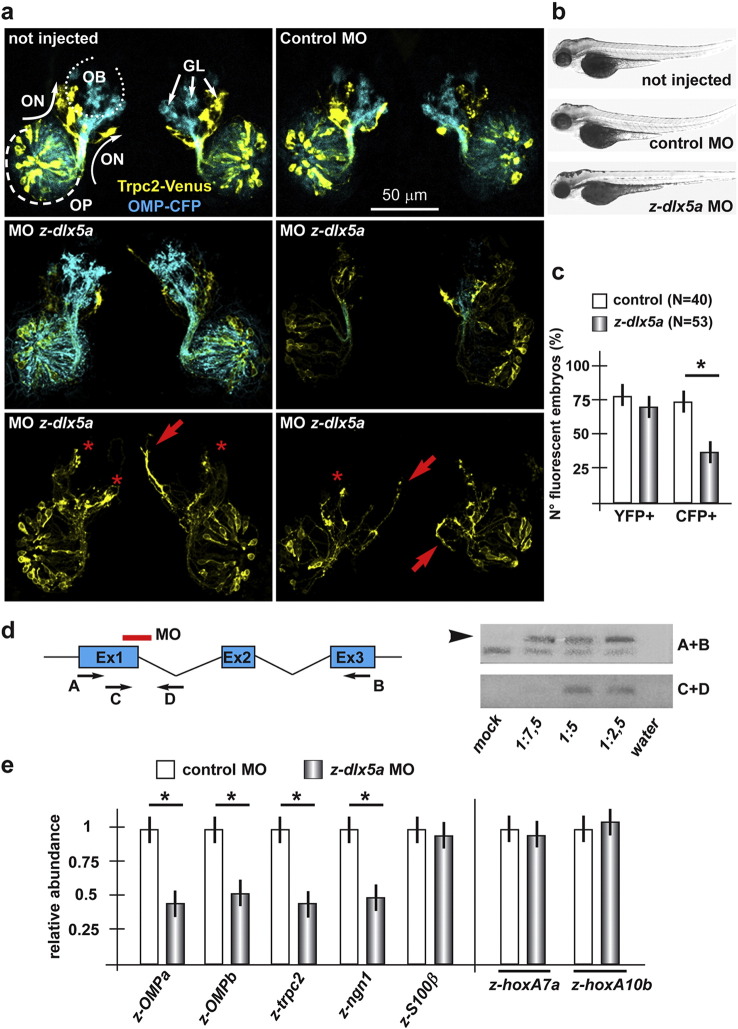
Depletion of *z-dlx5a* in zebrafish embryos causes delayed olfactory differentiation. a. Confocal stacked images of *Trcp2::Venus* (left, yellow fluorescence) and *OMP::CFP* (right, cyan fluorescence) zebrafish embryos not injected (left), injected with a control MO (right), or injected with anti-*z-dlx5a* MO (bottom panels), taken at 72 hpf. Arrows indicate the normal axonal pathway in the control embryos. Asterisks indicate the regions of reduced fluorescence intensity. b. Whole-mount bright field micrographs of injected embryo, showing an overall normal embryonic morphology and growth rate in the injected embryos, compared to the non-injected ones. c. Percentages of embryos showing YFP or CFP fluorescence, over the total of examined ones, comparing not-injected, control injected and MO injected ones. d. RT-PCR analysis on RNA extracted from anti-*z-dlx5a* MO-treated and control embryos, showing that the MO efficiently generates an inactive splice-variant form of the endogenous mRNA. A scheme of the *z-dlx5a* gene (Ex1–Ex2–Ex3), the positions of the *z-dlx5a* MO and the position of the PCR primers (A–D) are reported on the left. e. (on the left) Quantification of the olfactory differentiation phenotype by Real-Time qPCR for differentiation-related mRNAs in a sample of the embryonic heads of MO-injected embryos (grey bars). Embryos injected with control MO were used for comparison (open bars). Normalization is carried out relative to control samples, made = 1. (on the right) Relative abundance of *z-hoxA7a* and relative abundance of *z-hoxA10b* mRNAs in whole embryos injected with *z-dlx5a* MO (grey bars), to monitor developmental progression and exclude a generalized delay.

To deplete *z-dlx5a*, the fish ortholog of mammalian *Dlx5* (Ellies et al., 1997; MacDonald et al., 2010; Quint et al., 2000), we used a combination of two morpholino oligos (MO), as previously reported (Garaffo et al., 2013): the first designed to anneal with the exon1–intron1 splice junction, expected to interfere with the splicing of the z*-dlx5a* primary transcript and to result in a premature stop codon upstream of the homeodomain; the second designed to anneal with the ATG start codon and expected to inhibit the translation of the z-*dlx5a* mRNA (sequences in Suppl. Table III). The depletion of z-*dlx5a* was previously shown to cause axon misorientation and altered glomeruli formation in the MO-injected zebrafishes (Garaffo et al., 2013). Here we focused on OE differentiation at 72 hpf. First we verified that the splice-variant MO caused efficient intron 1 retention of the endogenous *z-dlx5a* mRNA (Fig. 4d). Injection of the control MO in the eggs from *OMP::CFP × Trpc2::YFP* strains did not cause significant reduction in the number of embryos that were CFP + (observed frequency 72%, expected frequency 67%, total analysed N = 40). The expected frequency represents the fact that we examine single-positives from CFP + × YFP + breeds, and the double-negative are then eliminated. Upon injection of the *z-dlx5a* MO, about 70% of the CFP + embryos showed a clear reduction of the CFP signal intensity (Fig. 4a–c), while injection of the same in the *Trpc2::Venus* strain yielded 72% of YFP + embryo (78% in the control injected), in which the YFP + fluorescence was seldom reduced (< 10% of YFP +) (Fig. 4a–c). The reduced fluorescence intensity of the OMP::CFP reporter and the reduced number of CFP + embryos counted after injection suggest a delayed differentiation of the OMP-type olfactory neuron. In contrast, the differentiation of the Trpc2-type neurons seemed minimally unaffected. To better document this, we measured the abundance of endogenous *z-OMPa*, *z-OMPb*, *z-Trpc2*, *z-ngn1* and *z-S100β* mRNAs in the head-piece of injected embryos, by Real-Time qPCR. As Trpc2 and OMPa/b are early markers for the differentiation of VNO-type and OE-type neurons, respectively, and since these mRNAs are not expressed in other embryonic territories, they are well suited to monitor overall olfactory epithelium differentiation. With the exception of *z-S100β*, all these differentiation markers showed a 40–60% reduced expression in the injected embryos, indicating a true differentiation delay (Fig. 4e). Overall, embryonic development and morphology were not significantly changed, revealed by visual observation. As a further control for the general developmental progression, the abundance of endogenous *z-hoxA7a* and *z-hoxA10b* did not significantly change upon MO injection (Fig. 4e). Thus, reduced *z-dlx5a* level does not affect general embryonic development.

In conclusion, the depletion of *z-dlx5a* in the fish embryos causes a differentiation delay on the OMP-type ORN and a general alteration of trajectory and connectivity of the olfactory axons (Garaffo et al., 2013), phenotypes fully recapitulating all main aspects of the *Dlx5*^−/−^ phenotype in the mouse (Levi et al., 2003; Long et al., 2003; Merlo et al., 2007).

### 3.6 Depletion of miR-9 and miR-200-class in zebrafish results in delayed ORN differentiation

**Fig. 5:**
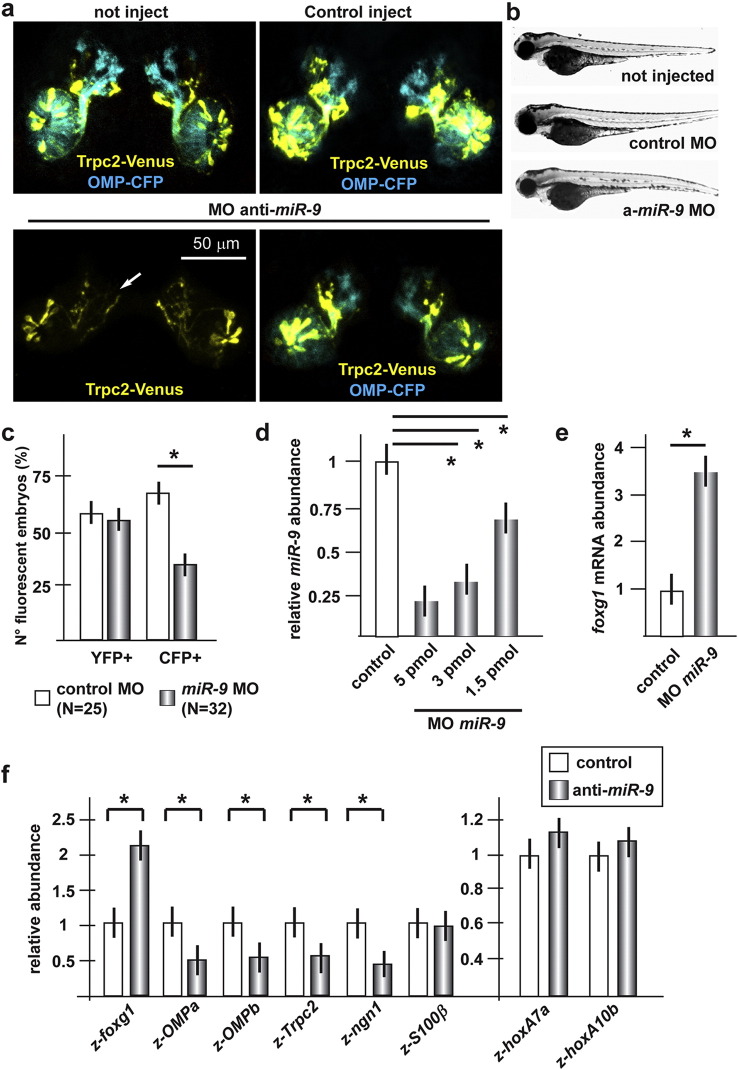
Depletion of *z-miR-9* in zebrafish embryos causes delayed olfactory differentiation. a. Micrographs of *Trcp2::Venus* (left, yellow fluorescence) and *OMP::CFP* (right, cyan fluorescence) zebrafish embryos injected with control (top panels) or anti-*z-miR-9* (bottom panels) MOs. The control MO did not cause any significant alteration. Arrows indicate the normal axonal pathway in the control embryos. Asterisks indicate the regions of reduced fluorescence intensity. b. Whole-mount bright field micrographs of injected embryo, showing a normal embryonic morphology and growth rate. c. Proportion of embryos showing either YFP or CFP fluorescence, upon injection of control or *z-dlx5a* MO. A significant loss of CFP + embryos is detected. d. Quantification of endogenous *miR-9* in zebrafish embryos injected with control or anti-*z-miR-9* MOs, by Real-Time qPCR. Results show efficient depletion. e. Quantification of endogenous *z-foxg1* mRNAs in zebrafish embryos injected with anti-*z-miR-9* MO, by Real-Time qPCR. Results show that depletion of *miR-9* causes a significant increase of the *z-foxg1* mRNA. f. Quantification of developmental markers, by Real-Time pPCR, in zebrafish embryos injected with anti-*miR-9* MOs, relative to control MO injected embryos. Samples were collected 72 hpf. Results are shown relative to the control injected samples, made = 1. The relative abundance of *z-hoxA7a* and relative abundance of *z-hoxA10b* mRNAs were determined, to monitor progression of development and exclude a generalized delay.

To functionally demonstrate a role of *miR-9* and *miR-200*-class for olfactory development, and the involvement of Foxg1 in this regulation in vivo, the zebrafish model was again used. The sequence of miR-9 and mi-200-class shows a high degree of identity between mouse and zebrafish (95% to 100%), as well as high similarity in their expression territories in early embryos ((Choi et al., 2008; Wienholds et al., 2005) and public databases). The knock-down of *miR-9* in zebrafish embryos, via injection of a MO previously shown to be specific and effective (Leucht et al., 2008) (sequence in Suppl. Table III), led to a significant and dose-dependent reduction of the endogenous *miR-9*, relative to control-injected ones, accompanied by a 3.5-fold increase of the endogenous z-*foxg1* mRNA (Fig. 5d, e). Upon injection of the anti-*miR-9* MO, only approximately 45% of the embryos were found to be CFP + (72% in the control injected), and in these we observed a clear reduction of the CFP + signal. On the contrary, we counted a nearly normal percentage of YFP + embryos (63%), and these seldom showed reduced fluorescence (Fig. 5a–c). The majority of anti*-miR-9* injected embryos displayed a normal placode organization, a normal pattern of olfactory axon fasciculation, extension and connectivity, and normal glomeruli formation. These data indicate that the depletion of *miR-9* results in a delayed or absent differentiation of the OMP + type ORN, with only a minimal effect of the Trpc2 + type neurons, and minimal consequences on axon/glomeruli organization.

To further confirm the role of *miR-9* in ORN differentiation, we carried out quantitative measurements of the mRNA levels of the OE differentiation genes, which include *z-foxg1*, *z-OMP-a*, *z-OMP-b*, *S100β* and *z-ngn1*, in the dissected frontonasal piece of fish embryos injected with MOs anti-*miR-9*. While the *z-foxg1* mRNA increased, the level of other differentiation markers decreased, with the exception of *S100β* (Fig. 5f). To exclude a generalized effect on development, we detected the mRNA abundance of *z-hoxa-7a* and *z-hoxa-10b*, two genes controlling general patterning and body plan, whose expression is strongly dependent upon embryonic age, and found that their abundance remains nearly constant (Fig. 5f). This provides an indication that the differentiation delay observed upon depletion of *miR-9* is specific for the olfactory and anterior brain regions.

**Fig. 6:**
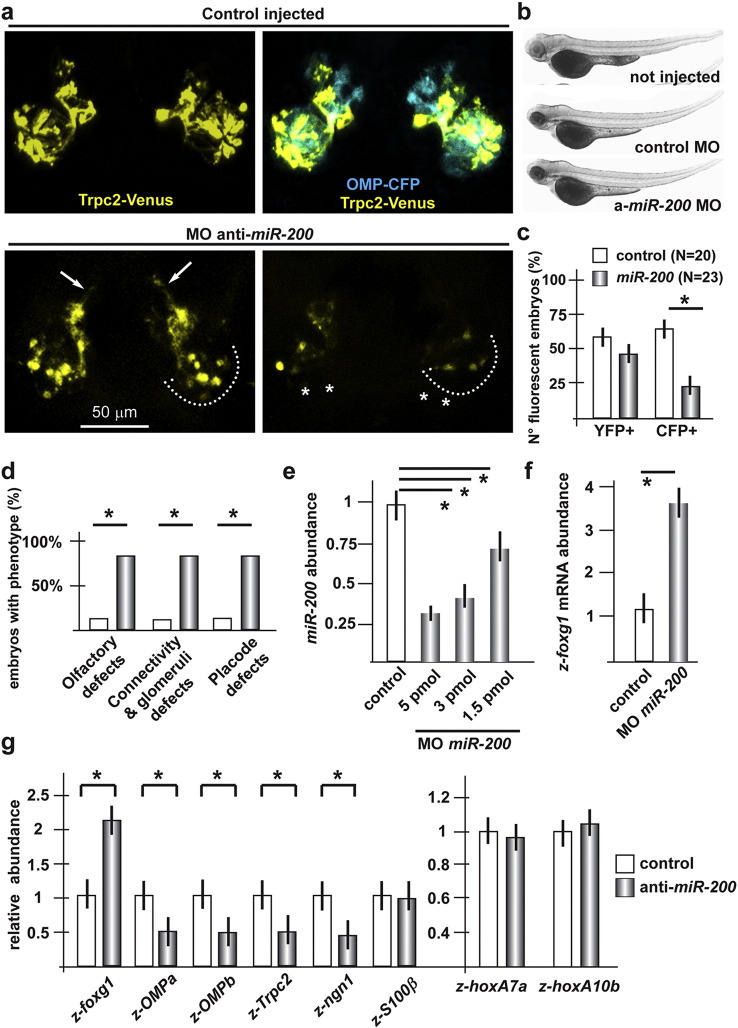
Depletion of *z-miR-200-*class in zebrafish embryos causes delayed olfactory differentiation. a. Micrographs of *Trcp2::Venus* (left, yellow fluorescence) and *OMP::CFP* (right, cyan fluorescence) zebrafish embryos not injected (left), or injected with control MO, or injected with anti-*z-miR-200* class (right panels) MO. The control MO did not cause significant alterations. Asterisks indicate the regions of reduced fluorescence intensity. b. Whole-mount bright field micrographs of injected embryo, showing normal embryo morphology and growth rate. c. Proportion of embryos showing either YFP or CFP fluorescence, upon injection of control or *z-dlx5a* MO. A significant loss of CFP + embryos is detected. d. Proportions of embryos showing either placode disorganization, olfactory axon mistargeting, or both (last bars) after injection of control or *z-miR-200 class* MOs. e. Quantification of endogenous *miR-200 class* in embryos injected with control or anti-*z-miR-200* MOs, by Real-Time qPCR. Results show a significant decrease of *miR-200* abundance in the injected embryos. f. Quantification of endogenous *z-foxg1* mRNA in zebrafish embryos injected with anti-*z-miR-9* MO, by Real-Time qPCR. Results show that depletion of *miR-200* causes a significant increase of *z-foxg1* mRNA abundance. g. Quantification of developmental markers, by Real-Time pPCR, in zebrafish embryos injected with anti-*miR-200* class MOs, relative to control MO injected embryos, set = 1. The abundance of *z-hoxA7a* and abundance of *z-hoxA10b* were also determined and used as in Fig. 5.

*miR-200a*, *miR-200b*, *miR-141* and *miR-429* share the same seed sequence and likely target the same mRNAs; for this reason they are grouped in a single miR class (named *miR-200*-class). We depleted the *miR-200* class in fish zygotes, by injecting a mix of anti*-miR-200* MO previously described and found to efficiently down-modulate several miR of the class-200 and to affect ORN differentiation (Choi et al., 2008). Upon injection of the *anti-miR-200* MO mix, only about 24% of examined embryos turned out CFP + (vs. 70% in the control injected), and in these we observed a clear reduction/absence of the CFP signal. The trajectory and glomeruli of CFP + fibres could not be examined. In the same experiment, about 45% of the examined embryos were found to be YFP + (vs. 60% in the control injected), and in these we often observed a clear reduction in the YFP signal intensity (Fig. 6a–c). In addition, we also observed defects of olfactory placode organization, alterated trajectory and reduced glomeruli formation of the YFP + fibres (Fig. 6d). Real-Time qPCR on anti-miR injected embryos demonstrated a dose dependent depletion of endogenous *miR-200* class, compared to control injected ones, accompanied by a 3.5-fold increase of *z-foxg1* mRNA in the same embryos (Fig. 6e, f).

Quantitative measurements of the mRNA levels of the OE differentiation genes *z-foxg1*, *z-OMPa*, *z-OMPb*, *S100β* and *z-ngn1*, in the frontonasal piece of fish embryos injected with MOs anti-*miR-200*-class revealed increased levels of *z-foxg1* mRNA, while the other differentiation markers decreased, with the exception of *S100β* (Fig. 6g). The abundance of *z-hoxa-7a* and *z-hoxa-10b* mRNAs did not greatly change, indicating that the differentiation delay observed upon depletion of *miR-200*-class is specific.

### 3.7 Depletion of miR-9 and miR-200-class in zebrafish results in altered GnRH neuron genesis and position

**Fig. 7:**
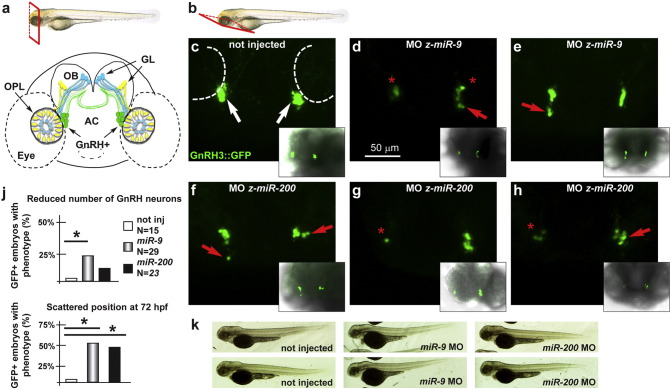
Depletion of *z-miR-9* and *z-miR-200-class* in zebrafish embryos affects differentiation and migration of GnRH + neurons. a. Scheme showing the positions of the GnRH3::GFP + neurons (green), relative to the OPL, the OB and the olfactory nerves (yellow and blue), in a frontal view. The anterior commissure is shown at the basis of the OB. On the top is the scheme illustrating the view plane (frontal). AC, Anterior Commissure; GL, Glomeruli; OB, Olfactory Bulb; OPL, Olfactory Placode. b. Scheme illustrating the view plane (ventral) used for the fluorescent images. c–h. Micrographs of *GnRH3:GFP* zebrafish embryos not injected (c), injected with anti-*z-miR-9* MO (d, e), or with anti-*z-miR-200* class (f–h). Insets show low-magnifications merged micrographs, for reference. In not injected or control MO-injected embryos, no significant alteration was observed. White arrows indicate the normal position of the GFP + neurons. Red arrows and asterisks indicate, respectively, mispositioned GFP + neurons and loss of neurons. j. Quantification of the observed phenotypes (top, reduced number of neurons; bottom, scattered position) expressed as percent of the injected GFP + embryos showing the indicated phenotype, over the total number of GFP + embryos examined, expressed as %. k. Whole-mount bright field micrographs of control and injected embryo, showing normal embryonic morphology and growth.

To determine whether *miR-9 and miR-200-*class play a role in GnRH neuronal differentiation and migration, we used the *GnRH3:GFP* transgenic zebrafish strain, in which the GFP reporter is expressed under the transcriptional control of a fragment of the z-*GnRH3* promoter. The GnRH3-GFP + neurons have been widely characterized, and they consist in a population of terminal-nerve associated neurons (see Fig. 7a), thought to correspond to the mammalian hypothalamic neurons of olfactory origin (Abraham et al., 2008, 2009, 2010; Wang et al., 2010). These neurons begin expression of GnRH around 24–30 hpf, and no other marker is known to specifically identify them, prior to GnRH. Their migration velocity has been estimated to be around 1 µm/hr (Y. Zhao et al., 2013), e.g. quite slow, while neurite extension is relatively faster.

We used the same MOs indicated above to deplete *miR-9* and *miR-200* class in *GnRH3::GFP* zygotes, and examined the effect on the number and position of the GFP + neurons associated to the terminal nerves, between 36 and 72 hpf. Counting was done on higher magnification (40 ×) Z-stack images on fixed 72 hpf embryos, as previously indicated (Y. Zhao et al., 2013) (see Materials & Methods section, and Suppl. Fig. 5); the position was determined by confocal imaging of fixed embryos at 60 and 72 hpf; migration was assessed by time-lapse video recording on live embryos, between 36 and 52 hpf. In control embryos, we counted an average of 13 (+/− 2) GnRH3::GFP + neurons/embryo at 72 hpf, while in *miR-9* and *miR-200* MO injected embryos the average number was, respectively, 5 (+/− 1) and 6 (+/− 1) (Suppl. Fig. 5). We arbitrarily established 8 neurons (− 35% with respect to the control) as threshold-value to score for the presence of the phenotype “reduced number”. Compared with the control injected embryos (N = 15), the depletion of *miR-9* (N = 29) resulted in a higher fraction of GFP + embryos with this phenotype (42% of cases. Fig. 7). In addition, in several cases the GFP + neurons appeared mispositioned or scattered; we termed this phenotype “scattered position” and detected this condition in 54% of the cases (Fig. 7). Similarly, the depletion of *miR-200*-class (N = 23) resulted in a reduced number of GFP + neurons in 22% of GFP + embryos with the phenotype “reduced number” and 50% of the cases showing the phenotype “scattered position” (Fig. 7).

To complement the previous (static) data with live images of the migrating GnRH3 neurons, we carried out few time-lapse video recordings on untreated (4) and z-*miR-200*-class MO injected (4) embryos at earlier ages (36–52 hpf), in order to observe the first appearance of these neurons. During this time-window, migration of GFP + cells turned out to be quite slow (1 µm/hr), in agreement with published data (Abraham et al., 2008), thus this experiment would require a recording time incompatible with maintaining the embryos alive. Furthermore, with such a slow migration, differences are unlikely to be detected. Instead, we could easily monitor the number and position of early GFP-expressing neurons, and noted that upon depletion of *miR-200* class they appear reduced in number but normally clustered. This could be taken as an indication that the “reduced number” phenotype is due to reduced neurogenesis of these cells, while the “scattered position” phenotype is acquired later and therefore possibly due to altered migration. Representative videos are provided as Supplementary Material.

Overall, these phenotypes recapitulate that described upon depletion of the Kallmann disease-genes ortholog *z-kal1a/b* (Whitlock et al., 2005; Yanicostas et al., 2009), *nelf* (Palevitch et al., 2009) and *z-fgfr1a* (Garaffo et al., 2013), and provide the first evidence of a role of specific miRs in GnRH neuron development.

### 3.8 Exogenous expression of z-foxg1 mRNA results in reduced ORN differentiation

The forkhead transcription factor gene *Foxg1* is expressed in the forebrain and OE of early mouse (Suppl. Fig. 4) and zebrafish embryos, and plays a role in maintaining olfactory progenitor cells in a “stem state” and prevent their premature differentiation (Duggan et al., 2008; Kawauchi et al., 2009b; Manuel et al., 2011). We raised the hypothesis that, in the absence of *Dlx5* and reduced levels of *miR-9* and -*200-*class, Foxg1 protein level is increased due to higher stability/translation of the *Foxg1* mRNA. In turn, an excess of Foxg1 could maintain ORNs in a progenitor-like state and prevent their timely differentiation. We set forth to test this hypothesis using zebrafish embryos.

We previously verified that the depletion of *miR-9* and *miR-200-class* in zebrafish embryos leads to higher level of *z-foxg1* mRNA (no Ab efficiently recognizes the z-foxg1 protein). We injected anti-*miR-9* and anti-*miR200* (or control) MOs in WT zygotes, then at 48 hpf we extracted total-RNA from these and carried out Real-Time qPCR analyses. *z-foxg1* mRNA level increased by three-folds when either *miR-9* or *miR-200*-class were depleted (Figs. 5e and 6f).

**Fig. 8:**
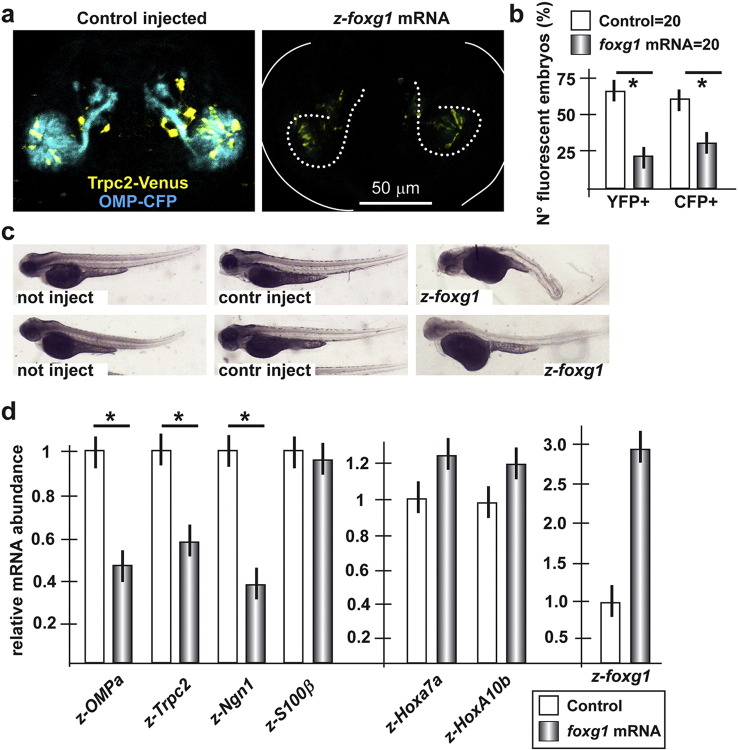
Injection of *z-foxg1* mRNA in zebrafish embryos causes delayed differentiation of olfactory neurons. a. Micrographs of double-positive *Trpc2::Venus*; *OMP::CFP* zebrafish embryos injected with control irrelevant mRNA (left) or with *z-foxg1* mRNA (right), taken at 72 hpf. Arrows indicate the normal axonal pathway in the control embryos, asterisks indicate the regions of reduced fluorescence intensity, dotted lines outline the placode and the main axonal bundle. b. Proportion of embryos showing either YFP or CFP fluorescence, upon injection of control (irrelevant) mRNA (open bars) or with *z-foxg1* mRNA (grey bars). A significant loss of both YFP and CFP + embryos is detected. Asterisks indicate p < 0.005. c. Whole-mount bright field micrographs of injected embryo, showing slightly altered embryonic morphology and nearly normal growth rate. d. (left) Quantification of endogenous *z-OMPa*, *z-Trcp2*, *z-ngn1* and *z-S100β* mRNAs in embryos injected with *z-foxg1* mRNA, by qPCR. Values are normalized against the control injection, set = 1. The over-expression of *foxg1* causes a significant decrease of these mRNAs, with the exception of *z-S100β*. (right) Quantifications of *z-hoxA7a* and *z-hoxA10b* mRNAs in WT embryos injected with anti-*z-foxg1* mRNA, relative to control injected embryos, used as in Fig. 5. Quantification of *z-foxg1* mRNA was used to verify the injection.

Next, we in vitro transcribed the *z-foxg1* mRNA, injected this in *OMP::CFP +* and *Trpc2::Venus*+ 1-cell zygotes and 72 hpf examined ORN differentiation and axon extension/trajectory. Higher doses (20 ng) of *z-foxg1* mRNA caused severe developmental anomalies, including small size, microcephaly, ocular dysmorphology, etc. … (data not shown). Nevertheless, lower doses (5–7 ng) affected general embryonic development less severely, and resulted in a reduced frequency of reporter-positive embryos (22% for Venus, 28% for CFP, N = 20) and a strongly reduced expression of both CFP and Venus reporters (Fig. 8a,b). In those few cases in which fluorescence was detected, we observe only mild effects of the injected mRNA on olfactory axon trajectory and fasciculation (Fig. 8a). To confirm that reduced fluorescence intensity truly reflects altered gene expression and delayed differentiation, and to rule out generalized effects on embryonic development, we examined the relative abundance of the olfactory differentiation-related mRNAs *z-OMPa, z-OMPb, z-Trpc2, z-ngn1* and *z-S100β* upon injection of *z-foxg1* mRNA in WT zygotes, by Real-Time qPCR. The results show that all the differentiation-related mRNAs (examined) were significantly down-regulated (about 70-80% reduction), compared to the control, with the exception of *z-S100β*. As control for general developmental progression, the abundance of z-*hoxa7a* and *z-hoxa10b* mRNAs was mildly increased (Fig. 8d). These results indicate that higher expression of *foxg1* has similar effects as *Dlx5*, *miR-9* and -*200* depletions on olfactory differentiation, in vivo.

## 4 Discussion

The results presented here indicate that loss of *Dlx5* causes a down-modulation of *miR-9* and of *miR-200-*class, which results in the over-expression of the Foxg1 protein. Starting from profile data obtained from a mouse model of Kallmann syndrome, we functionally examined this pathway in zebrafish showing that *miR-9* and *miR-200-*class are required for normal differentiation of the ORNs, for the extension and connectivity of the olfactory axons, and for the migration of the GnRH neurons from the nasal primordium to the forebrain. Then we determined that altered levels of Foxg1 are also associated with delayed/reduced differentiation of the ORNs, and altered olfactory axons trajectory.

### 4.1 The role of miR in ORN differentiation and axonal trajectory

Olfactory development is often used in studies on the cellular and molecular bases of neuronal differentiation, migration and axon targeting. Research has focused on the identification of protein-coding genes that control ORN differentiation and axon targeting, and several mutant mouse strains show specific phenotypes affecting these processes. Conversely, little information is available on the role of specific miRs and other non-coding RNAs, in the same processes. An indication that miR are needed for olfactory development comes from the results of the conditional deletion of *Dicer* using the *OMP-Cre* or the *Foxg1-Cre* deleter mice (Choi et al., 2008). In these models, all mature miRs are depleted, in the *Cre* + cells. Mice in which *Dicer* was depleted using *OMP-cre* show delayed ORN differentiation and survival, while showing little or no effect on axon guidance, glomerulus formation and topography. Accordingly, GnRH neuron migration was found to be nearly normal. It is important to notice that he *OMP* promoter drives *Cre* expression in OE and VNO neurons from the age E14.5 onward, i.e. when olfactory connections are formed and GnRH neuron have reached the forebrain. Thus, this model fails to reveal the role of miRs in olfactory axon guidance and connectivity, as well as in GnRH neuron migration, events that occur at a much earlier age. Conversely, mice in which *Dicer* is removed with *Foxg1-Cre* show a severely impaired ORN differentiation and reduced cell survival (Choi et al., 2008). Since *Foxg1* is expressed much earlier than OMP in placodal cells, it can be concluded that miRs are required at an early stage of development, and not late. However, later aspects of olfactory development, such as full ORN differentiation, axonal connection and GnRH migration, were not examined. Furthermore, since the disruption of *Dicer* prevents the maturation of all miRs, the *Dicer* conditional mutant models lack specificity.

The sequence-based specificity of miR function is the key to begin decipher the molecular pathway underlying olfactory development. The most abundant miRs expressed in the developing mouse OE are: the *miR-200*-class (-*200a,* -*200b,* -*200c,* -*141* and -*429*), *miR-199, miR-152, miR-214, miR-205, miR-183, miR-182* and *miR-96* (Choi et al., 2008). However, when Dlx5 is absent we do not see a generalized reduction of miR expression, rather the downmodulation of specific ones. This might explain the fact that in the OE of *Dlx5*^−/−^ embryos we see a relatively mild differentiation phenotype compared to that of the *Dicer^flox^*; *Foxg1::Cre* mice or the anti*-miR-200-*class morphants (Choi et al., 2008). In this work we define the role of *miR-9* and *miR-200-*class in the development of the olfactory system, with functions ranging from ORN differentiation to axon guidance, glomerulus formation and GnRH neuron migration. We also show that *miR-9* and *miR-200*-class target (amongst others) the *foxg1* mRNA, through which they likely exert their functions. Previous results in which zebrafish embryos were injected with anti-*miR-200* class MOs found a delayed ORN differentiation, but axonal organization and GnRH neuron migration was not assessed (Choi et al., 2008). Examining olfactory development more thoroughly we now can implicate the *miR-9* and *miR-200-class* networks in a more complex phenotype reminiscent of the Kallmann syndrome (see below).

### 4.2 miR-9 and miR-200 mediate the Dlx5-Foxg1 cascade

*Foxg1* is a winged helix transcription factor, member of the fork-head family, expressed in the early developing forebrain and peripheral olfactory system. In the forebrain, the loss of *Foxg1* causes premature lengthening of progenitor cell cycles and increased neurogenic divisions, leading to a severe brain hypoplasia (Manuel et al., 2011). It has been proposed that these proliferation defects could be a secondarily caused by altered expression of *FGF8* and *BMPs* in the forebrain of *Foxg1* null mutants. By the generation and analysis of *Foxg1^+/+^* ↔ *Foxg1^−/−^* chimaeras, Foxg1 appears to control forebrain progenitor proliferation cell autonomously (Manuel et al., 2011).

*Foxg1* is also expressed in early progenitor cells of the OP, and later the expression becomes restricted to the ventro-lateral OE and VNO, in cells within the basal compartment of the OE, the location where OE stem and progenitor cells are known to reside (Kawauchi et al., 2009b). In early *Foxg1*^−/−^ embryos a small number of progenitors are initially specified but show reduced proliferation and differentiation. Older *Foxg1*^−/−^ embryos show no recognizable OE, VNO and OB (Duggan et al., 2008). BrdU pulse-chase labelling of *Sox2*-expressing stem cells indicated that these cells are delayed or halted in their development, in the absence of *Foxg1* (Kawauchi et al., 2009a,b), suggesting that the proliferation and/or subsequent differentiation of *Sox2*+ stem cells in the OE is regulated by *Foxg1*. Thus *Foxg1* is required for the development of the central and peripheral olfactory system, to maintain progenitors in a proliferative state (Duggan et al., 2008).

The 3′ UTR of tetrapod and zebrafish *Foxg1* mRNAs hosts *miR-9* and *miR-200* target sequences. Indeed Foxg1 has been experimentally shown to be negatively regulated by *miR-9*. The mouse *miR-9* targets *Foxg1* mRNAs for proper generation of Cajal–Retzius neurons in the medial pallium (Shibata et al., 2008). *miR-9* expression is medio-laterally graded, being most intense in the cortical hem; it contrasts with the *Foxg1* expression in a reciprocal gradient. *miR-9* over-expression in developing forebrain at E11.5 resulted in ectopic Reelin + cells over the cortex beyond the marginal zone, while conversely the inhibition of endogenous *miR-9* function caused the regression of *Wnt3a* positive cortical hem and reduction of *Reelin*+, *p73*+ and *NeuroD1*+ cells (Shibata et al., 2008). Thus, a fine modulation over the level of Foxg1 protein is required for efficient and timely differentiation of specific subset of cortical neurons.

Here we show that mouse and fish *foxg1* mRNA is a target of *miR-9* and *miR-200* class, both of which are down-modulated in the *Dlx5* null embryonic OE. We also show that Dlx5 promotes expression of *miR-9* and *miR-200* class, thereby tends to repress Foxg1 protein translation. Hence, the most likely scenario is that in the absence of Dlx5 the Foxg1 protein persists for a longer time or at increased level, preventing progenitor cells from efficiently exiting the progenitor state and initiate differentiation. This possibility is clearly consistent with the results reported by Shibata et al. (2008), in which they show that the depletion of *miR-9* resulted in abnormally high levels of Foxg1 proteins, and this caused a delayed differentiation of the Cajal–Retzius neurons in the cortex. It has also been shown that *miR-200* represses neural induction of human embryonic stem cells, via modulation of Pax6 and Zeb transcription factors (Du et al., 2013). We may propose that in neural stem cells, this regulation is carried out also via Foxg1. Our results reinforce the notion that the expression level of master transcription factors (such as Dlx5 and Foxg1 for ORN) must be precisely regulated in either directions to assure normal development.

Finally, Foxg1 and Emx2 have been shown to be key transcription factors that control the switch between gliogenesis and neuronogenesis, in stem cells (Brancaccio et al., 2010). Since Emx2 and Foxg1 are both expressed in the early olfactory progenitors, this observation is intriguing, as it is conceivable that an increased Foxg1 expression may imbalance this choice and lead to a retarded neuronogenesis, at the advantage of the generation of supporting cells, or other non-neuronal cell types present in the OE. We will address this issue in future works.

### 4.3 miRs, coding RNAs and the Kallmann syndrome

Part and current research on KS/nCHH has strongly focused on the identification of protein-coding genes. Conversely, there is little or no information as to weather miRs (and other non-coding RNAs) might be involved in the molecular pathogenesis of this disorders. One indications comes from the *SEMA3A* gene, which causes KS in human and a KS-like phenotype in mice (Cariboni et al., 2011; Hanchate et al., 2012); in retinal neurons the *SEMA3A* mRNA is regulated by *miR-124* and this regulation modulates the guidance response of these cells to the Sema3a signal (Baudet et al., 2011). Another indication comes from a study in zebrafish, showing a role of *miR-200*-class for olfactory development (Choi et al., 2008). Recently, *miR-200b* and *miR-429* have been linked to pituitary endocrine functions controlling ovulation and fertility in female mice (Hasuwa et al., 2013). Interestingly, we show here that miRs of the 200-class are involved in olfactory axons extension and GnRH neuron migration, thus suggesting that these miRs are indeed essential for male and female sexual maturation. Finally, the *lin28*/*let-7* miR system has been implicated in the maturation of the post-natal hypothalamus and induction of puberty, in a mouse model of CHH (Gaytan et al., 2013; Sangiao-Alvarellos et al., 2013).

In spite of this scattered knowledge, studies specifically addressing the expression and function of miR in GnRH neurons are needed to dissect their role in the genesis, migration and maturation of this specific cell type. At present, a systematic study to identify specific miRs relevant for early GnRH neuron development, in vitro or in vivo, has not been reported, as yet. Thus, our results provide the first evidence of the participation of *miR-9* and *miR-200*-class in these early events. We further link two transcription factors with the action of these miRs.

A pathway is emerging, yet to be fully unravelled. The possibility that Dlx5 may regulate aspects of the development of GnRH neurons was suggested by the finding that Dlx and Msx binding sites are present in the mammalian *GnRH* promoter (Givens et al., 2005). Furthermore, *Dlx5* is expressed in murine embryonic GnRH neurons, and in *Dlx5*^−/−^ mice the GnRH neurons fail to properly migrate (Levi et al., 2003; Merlo et al., 2007). These and the present results, however, do not fully demonstrate that Dlx5 is needed for GnRH neuron migration cell autonomously; rather the observed defects could be consequent of impaired olfactory axon elongation and/or connectivity, hence non-cell autonomous. These possibilities will have examined in further studies.

## Figures and Tables

**Table 1:** Enrichment of miR target sequences in the 3′ UTR of DEGs in *Dlx5^−/−^* OE.

	Genes	Expect	P
A. *miR-9* and *miR-200*-class in upregulated DEGs			
miR-141/200a	3	0.366999	0.00567807
miR-200bc/429/548a	3	0.575101	0.0191205
miR-9/9ab	3	0.704269	0.0323028

B. *miR-9* and *miR-200*-class in downregulated DEGs			
miR-141/200°	4	1.3212	0.0436507
miR-200bc/429/548a	3	2.07037	n.s.
miR-9/9ab	1	2.53537	n.s

C. Up *miR-200*	Up *miR-9*	Down *miR-200*	Down *miR-9*

Cyp26b1	Gabrb2	Akap6	Slc25a35
AI593442	Isl1	Elmod1	
Qk	Qk	Necab1	
Pitx2		Slc14a1	
Foxf2		Snap25	
