# Supplementary material for: The Dlx5 and Foxg1 transcription factors, linked via miRNA-9 and -200, are required for the development of the olfactory and GnRH system
Source: Mol Cell Neurosci. 2015 Sep;68:103–19. doi: 10.1016/j.mcn.2015.04.007 (PMC4604252; doi:10.1016/j.mcn.2015.04.007)

Supplementary Figure 1. Sequence and chromosomal locations of the miRs found to be down-modulated in the *Dlx5*-/- versus WT mouse OE. The miRs of the -200 class are separated in two sub-groups depending on the genome location.

miR-

clustered with other 16 miRNAs on chromosome 12

Mmu-miR-376a: 5'- **AUCGUAGAGGAAAAUCCACGU** – 3'

miR-9

miR-9.1 chromosome 3

miR-9.2 chromosome 13

miR-9.3 chromosome 7

Mmu-miR-9: 5'- **UCUUUGGUUAUCUAGCUGUAUGA** – 3'

miR-200

miR-200a
miR-200b clustered on chromosome 4
miR-429

miR-141
miR-200c clustered on chromosome 6

Mmu-miR-200a : 5' - **UAACACUGUCUGGUAACGAUGU**

Mmu-miR-141: 5' - **UAACACUGUCUGGUAAAGAUGG**

Mmu-miR-200b: 5' - **UAAUACUGCCUGGUAAUGAUGA** -

Mmu-miR-429: 5' - **UAAUACUGUCUGGUAAUGCCGU**

Supplementary Figure 2. Location of target sequences for *miR-9* and *miR-200*–class in the 3'UTR sequence of the mammalian (above) and of the zebrafish (below) *foxg1* mRNA. Note the conservation of the sites.

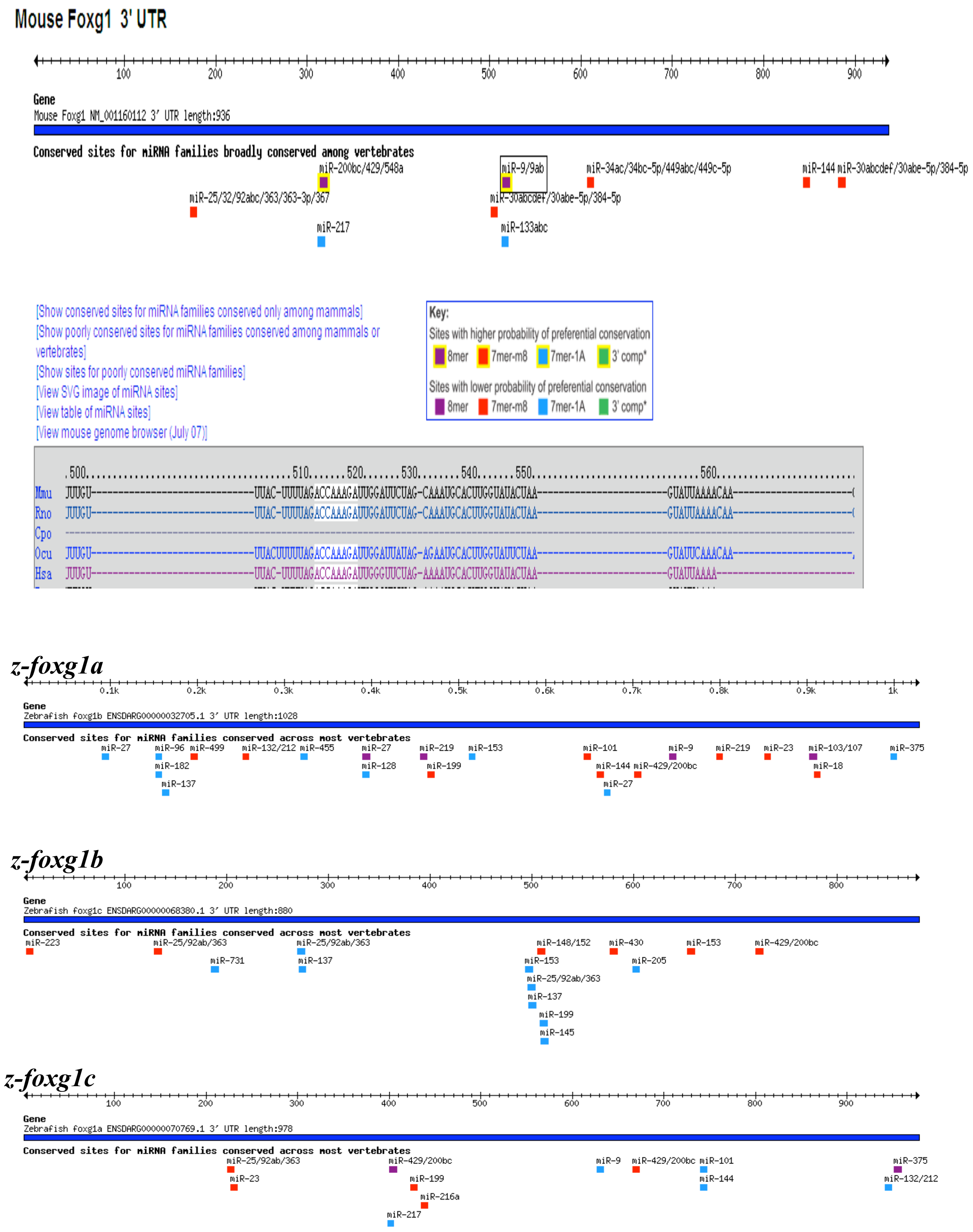

Supplementary Figure 3. **a.** Scheme illustrating the general position of the olfactory system with respect to the eyes, in the head of zebrafish embryos, in frontal view. GL, glomeruli; ON, Olfactory Nerves; OB, Olfactory Bulbs; OP, Olfactory Placode. **b,c.** Images of the organization and trajectory of the Trpc2::Venus (yellow fluorescence) and OMP::CFP (blue fluorescence) axons in the developing zebrafish embryo. The olfactory bulbs, olfactory nerves and glomeruli are indicated.

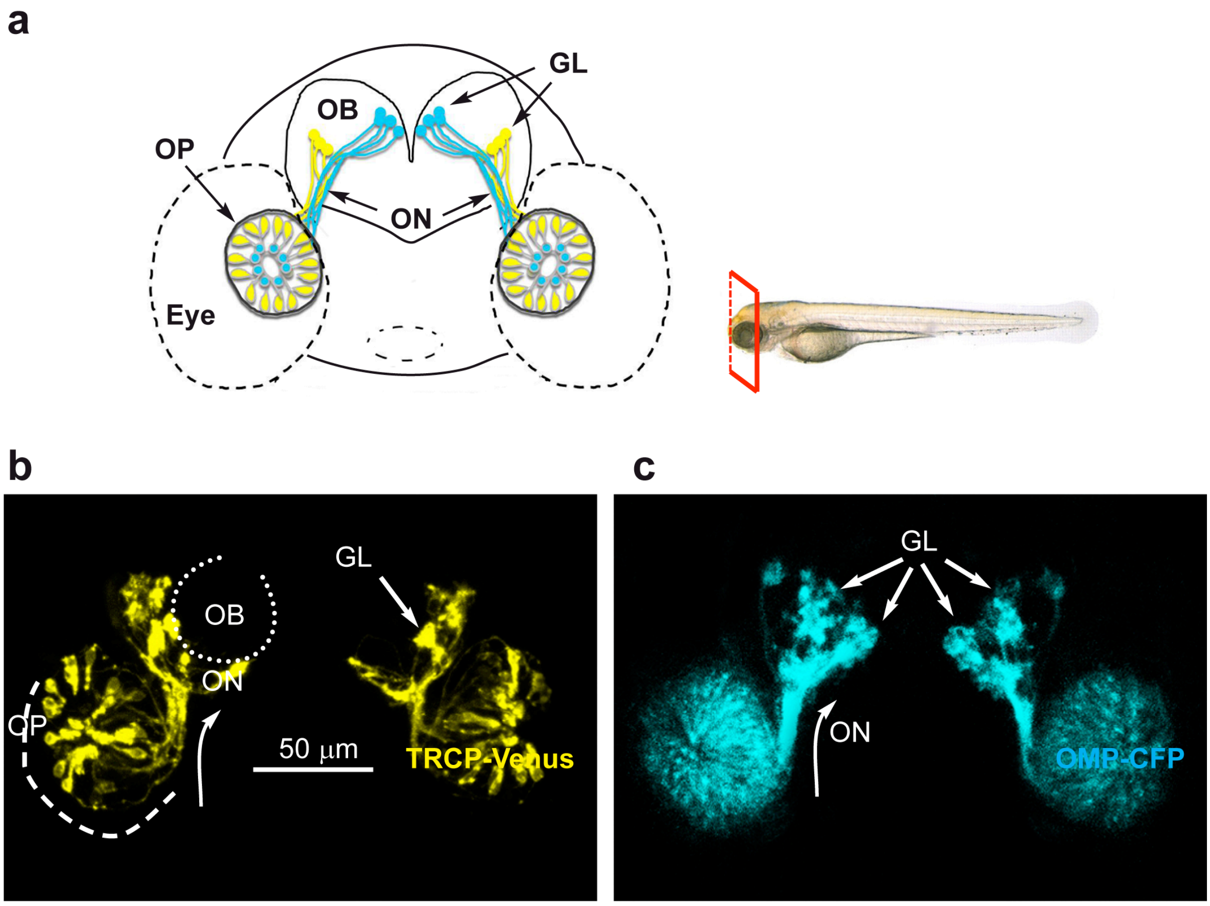

Supplementary Figure 4. Expression of *Foxg1* mRNA, detected by *in situ* hybridization, in the OE (red arrows) and forebrain (FB) of the mouse embryo, at the ages E11.5 (left) and E14.5 (right) (*from www.genepaint.org*).

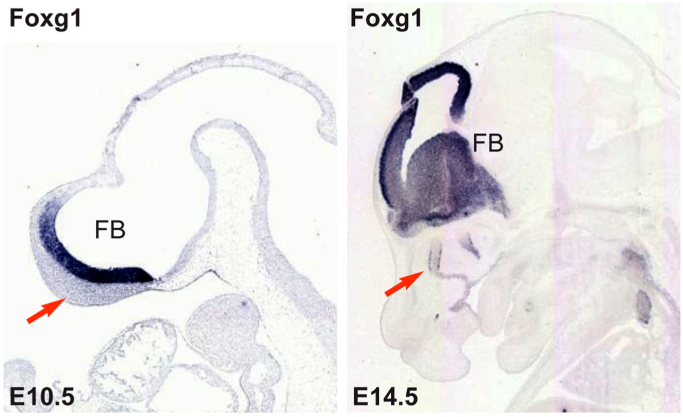

Supplementary Figure 5. High magnification of olfactory-associated GFP+ neurons upon injection of anti-*miR-9* and anti-*miR-200*-class MO in *GnRH3:GFP* zygotes. **a.** The view plane relative to the images below. **b**. Quantification of GnRH3::GFP+ neurons in untreated and MO-injected embryos. Asterisks:p<0.01. **c-e.** Representative micrographs of GFP+ cells in control (c), *miR-9* MO injected (d) or *miR-200* MO injected (e) embryos. Images on the left are low-maginification or the same on the right. Arrows indicate mispositioned cells; asterisks indicate reduced number or absent cells.

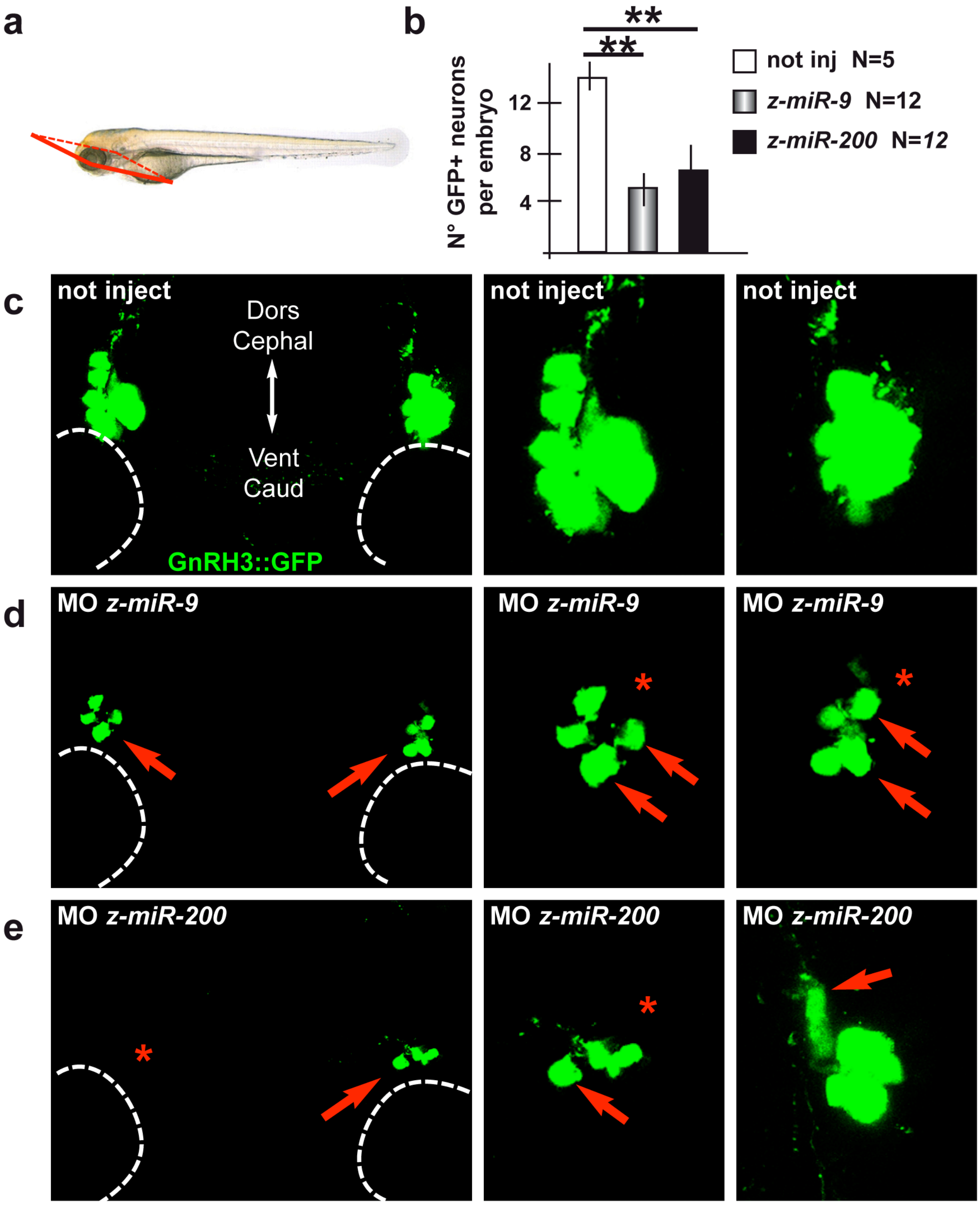

Supplement: Supplementary file 1 — Supplementary figures. [file mmc1.pdf]
