# Supplementary material for: The Dlx5 and Foxg1 transcription factors, linked via miRNA-9 and -200, are required for the development of the olfactory and GnRH system
Source: Mol Cell Neurosci. 2015 Sep;68:103–19. doi: 10.1016/j.mcn.2015.04.007 (PMC4604252; doi:10.1016/j.mcn.2015.04.007)
Supplement: Supplementary file 2 — Supplementary tables. [file mmc2.doc]

**Supplementary Table I.**

**Sequence of primers used for Real-Time qPCR on zebrafish RNA**

*z-OMPa* F GACATTCACCGAGGATCTG

R CGTCCTGCCGTTTCTGAC

*z-OMPb* F TTCACCCGCTGGAACATT

R GGTCATCAGGTGTGTGAGGTC

*z-trpc2* F TCCAAAGGCTGTTTTCTATCTTTT

R GGAGCTGAGTTACAGTTGAAGCA

*z-Ngn1* F TCACAGCGCATGAAATAACG

R GCTATCTCCGATGTGCATCC

*z-S100β* F CTCGTCGAAAGAAGGAGACAA

R gcttggtctttcacttgctca

*Z-HoxA7a* F gctggcgatctctgtaaagc

R ttttgatggtagcccctctg

*z-HoxA10b* F gagctaagggggtccactg

R cacttttggaatctcctgcttt

*z-foxg1a* F tccgtattaccgggagaaca

R aaactcaagttgtgtctgatggaa

*z-neurog1* F CAGGATTCTGCAAAACCTCAA

R cgatctccattgttgataacctt

*z-β-actin* F cgagctgtcttcccatcca

R tcaccaacgtagctgtcttctg

**Supplementary Table II.**

**Sequence of primers used for the analysis of immunoprecipitated human chromatin.**

*miR-9.3 10.57* For CCTAAAGCTCTGGCTCCTTCCTT

Rev ATCAGCTGAAGGCACCTCATTCTC

*miR-200a/b - miR-429* For TTCTTTGGGAGCTTGGAAGGC

Rev CAGGACCAGTTTCCAGCGAG

**Supplementary Table III. Sequences of MOs used for injection in zebrafish oocytes**.

| **Gene / Locus** | **Description** | ***D. rerio* homolog** | **Sequence** | **Morpholino targeting** |
| --- | --- | --- | --- | --- |
| *Dlx5* | distall-les homeobox 5 | *z-dlx5a* | 5'-CGAATACTCCAGTCATAGTTTGGAT-3' | ATG blocking |
|  |  |  | 5'-TTATATAACAACTAACCTTGCGGGC-3' | Ex1-In1 splice junct. |
| *miR-9* | microRNA-9 | *dre-mir-9-1 /*  *dre-mir-9-7* | 5'-TCTTTGGTTATCTAGCTGTATGA-3' | From  Leucht et al. 2008 |
|  |  |  |  |  |
| *miR-200 class* | mmu-mir-200b | *dre-mir-200a* | 5'-GTCATCATTACCAGGCAGTATTA -3' | From  Choi et al. 2008 |
|  | mmu-mir-141 | *dre-mir-141* | 5'-GCATCGTTACCAGACAGTGTTA-3' |  |
|  | mmu-mir-429 | *dre-mir-429* | 5'-ACGGCATTACCAGACAGTATTA-3' |  |

**Supplementary Table IV A. GeneOntology of miR-9 predicted targets, Biological Process**

| GOBPID | Pvalue | OddsRatio | ExpCount | Count | Size | Term |
| --- | --- | --- | --- | --- | --- | --- |
| GO:0045595 | 0,000329 | 1,474884 | 82,50382 | 112 | 772 | regulation of cell differentiation |
| GO:0034329 | 0,000614 | 2,719216 | 7,908397 | 18 | 74 | cell junction assembly |
| GO:0048666 | 0,000752 | 1,531002 | 56,64122 | 80 | 530 | neuron development |

**Supplementary Table IV B. GeneOntology of miR-9 predicted targets, Molecular Function**

| GOMFID | Pvalue | OddsRatio | ExpCount | Count | Size | Term |
| --- | --- | --- | --- | --- | --- | --- |
| GO:0003730 | 0,000351 | 5,426861 | 2,453333 | 9 | 23 | mRNA 3'-UTR binding |

**Supplementary Table IV C. GeneOntology of miR-9 predicted targets,** Cellular Components

| GOCCID | Pvalue | OddsRatio | ExpCount | Count | Size | Term |
| --- | --- | --- | --- | --- | --- | --- |
| GO:0030054 | 0,000336 | 1,562892 | 58,53819 | 84 | 554 | cell junction |
| GO:0030662 | 0,0006 | 3,235788 | 5,388895 | 14 | 51 | coated vesicle membrane |

**Supplementary Table V A. GeneOntology of miR-141/200a targets,** Biological Process

| GOBPID | Pvalue | OddsRatio | ExpCount | Count | Size | Term |
| --- | --- | --- | --- | --- | --- | --- |
| GO:0035295 | 3,57E-06 | 2,383933 | 19,37063 | 41 | 336 | tube development |
| GO:0006928 | 4,57E-06 | 1,879053 | 44,10277 | 74 | 765 | cellular component movement |
| GO:0045892 | 7,15E-06 | 1,980852 | 33,7833 | 60 | 586 | negative regulation of transcription, DNA-dependent |
| GO:0010629 | 8,58E-06 | 1,915352 | 37,81884 | 65 | 656 | negative regulation of gene expression |
| GO:0022008 | 1,8E-05 | 1,793741 | 45,83229 | 74 | 795 | neurogenesis |
| GO:0006366 | 1,91E-05 | 1,735824 | 53,21158 | 83 | 923 | transcription from RNA polymerase II promoter |
| GO:0051253 | 2,23E-05 | 1,895928 | 35,05161 | 60 | 608 | negative regulation of RNA metabolic process |
| GO:2000113 | 2,29E-05 | 1,865018 | 37,41529 | 63 | 649 | negative regulation of cellular macromolecule biosynthetic process |
| GO:0006357 | 2,46E-05 | 1,726868 | 52,75037 | 82 | 915 | regulation of transcription from RNA polymerase II promoter |
| GO:0051254 | 3,28E-05 | 1,776407 | 44,21807 | 71 | 767 | positive regulation of RNA metabolic process |
| GO:0000122 | 3,53E-05 | 2,074774 | 23,46383 | 44 | 407 | negative regulation of transcription from RNA polymerase II promoter |
| GO:0031327 | 3,93E-05 | 1,8094 | 39,66367 | 65 | 688 | negative regulation of cellular biosynthetic process |
| GO:0010557 | 4,19E-05 | 1,734831 | 47,79241 | 75 | 829 | positive regulation of macromolecule biosynthetic process |
| GO:0045944 | 4,95E-05 | 1,887329 | 32,11143 | 55 | 557 | positive regulation of transcription from RNA polymerase II promoter |
| GO:0045893 | 5,23E-05 | 1,759796 | 43,23801 | 69 | 750 | positive regulation of transcription, DNA-dependent |
| GO:0009790 | 5,51E-05 | 1,801981 | 38,51065 | 63 | 668 | embryo development |
| GO:0009888 | 5,77E-05 | 1,677458 | 54,01869 | 82 | 937 | tissue development |
| GO:0009890 | 6,67E-05 | 1,772421 | 40,35547 | 65 | 700 | negative regulation of biosynthetic process |
| GO:0010628 | 7,17E-05 | 1,713214 | 46,92765 | 73 | 814 | positive regulation of gene expression |
| GO:0010558 | 7,19E-05 | 1,782889 | 38,85656 | 63 | 674 | negative regulation of macromolecule biosynthetic process |
| GO:0045935 | 7,33E-05 | 1,700034 | 48,59952 | 75 | 843 | positive regulation of nucleobase-containing compound metabolic process |
| GO:0045934 | 8,07E-05 | 1,791114 | 37,41529 | 61 | 649 | negative regulation of nucleobase-containing compound metabolic process |
| GO:0048699 | 9,36E-05 | 1,727567 | 43,23801 | 68 | 750 | generation of neurons |
| GO:0060429 | 9,96E-05 | 1,863206 | 30,61251 | 52 | 531 | epithelium development |
| GO:0051172 | 0,00011 | 1,76843 | 37,81884 | 61 | 656 | negative regulation of nitrogen compound metabolic process |
| GO:0030182 | 0,000111 | 1,751889 | 39,43306 | 63 | 684 | neuron differentiation |
| GO:0051173 | 0,000121 | 1,66884 | 49,34898 | 75 | 856 | positive regulation of nitrogen compound metabolic process |
| GO:0045595 | 0,000128 | 1,699783 | 44,50632 | 69 | 772 | regulation of cell differentiation |
| GO:0035239 | 0,000135 | 2,264124 | 14,64327 | 30 | 254 | tube morphogenesis |
| GO:0030324 | 0,000147 | 2,983688 | 6,860431 | 18 | 119 | lung development |
| GO:0043009 | 0,000152 | 1,929734 | 24,96274 | 44 | 433 | chordate embryonic development |
| GO:0060487 | 0,000162 | 7,721042 | 1,268315 | 7 | 22 | lung epithelial cell differentiation |
| GO:0030323 | 0,000182 | 2,925036 | 6,975732 | 18 | 121 | respiratory tube development |
| GO:0009891 | 0,000188 | 1,630926 | 51,71266 | 77 | 897 | positive regulation of biosynthetic process |
| GO:0021537 | 0,000189 | 2,816631 | 7,609889 | 19 | 132 | telencephalon development |
| GO:0055023 | 0,000191 | #DIV/0! | 0,172952 | 3 | 3 | positive regulation of cardiac muscle tissue growth |
| GO:0061051 | 0,000191 | #DIV/0! | 0,172952 | 3 | 3 | positive regulation of cell growth involved in cardiac muscle cell development |
| GO:2000481 | 0,000191 | #DIV/0! | 0,172952 | 3 | 3 | positive regulation of cAMP-dependent protein kinase activity |
| GO:0051128 | 0,000202 | 1,626521 | 51,82796 | 77 | 899 | regulation of cellular component organization |
| GO:0009792 | 0,000208 | 1,898975 | 25,30865 | 44 | 439 | embryo development ending in birth or egg hatching |
| GO:2000026 | 0,000213 | 1,638144 | 49,40663 | 74 | 857 | regulation of multicellular organismal development |
| GO:0060479 | 0,000221 | 7,2376 | 1,325966 | 7 | 23 | lung cell differentiation |
| GO:0001654 | 0,000228 | 2,431135 | 10,95363 | 24 | 190 | eye development |
| GO:0030900 | 0,000257 | 2,31627 | 12,3949 | 26 | 215 | forebrain development |
| GO:0031328 | 0,000319 | 1,60726 | 50,90555 | 75 | 883 | positive regulation of cellular biosynthetic process |
| GO:0090344 | 0,000332 | 21,96016 | 0,403555 | 4 | 7 | negative regulation of cell aging |
| GO:0060428 | 0,000354 | 5,52008 | 1,844822 | 8 | 32 | lung epithelium development |
| GO:0060562 | 0,000363 | 2,184449 | 14,06677 | 28 | 244 | epithelial tube morphogenesis |
| GO:0021543 | 0,000365 | 3,240346 | 4,957958 | 14 | 86 | pallium development |
| GO:0055007 | 0,000403 | 3,888416 | 3,343739 | 11 | 58 | cardiac muscle cell differentiation |
| GO:0045596 | 0,000476 | 2,005939 | 17,92936 | 33 | 311 | negative regulation of cell differentiation |
| GO:0002009 | 0,000504 | 1,9985 | 17,98701 | 33 | 312 | morphogenesis of an epithelium |
| GO:0048745 | 0,000507 | 7,622769 | 1,095363 | 6 | 19 | smooth muscle tissue development |
| GO:0009892 | 0,000604 | 1,545733 | 56,26706 | 80 | 976 | negative regulation of metabolic process |
| GO:0055021 | 0,000656 | 5,787275 | 1,556568 | 7 | 27 | regulation of cardiac muscle tissue growth |
| GO:0016477 | 0,000671 | 1,709286 | 32,28438 | 51 | 560 | cell migration |
| GO:0035051 | 0,000675 | 3,180975 | 4,669705 | 13 | 81 | cardiocyte differentiation |
| GO:0030048 | 0,000681 | 4,904953 | 2,017774 | 8 | 35 | actin filament-based movement |
| GO:0055025 | 0,000729 | 49,32406 | 0,230603 | 3 | 4 | positive regulation of cardiac muscle tissue development |
| GO:0060284 | 0,000742 | 1,785242 | 26,05811 | 43 | 452 | regulation of cell development |
| GO:0031324 | 0,00075 | 1,556498 | 51,53971 | 74 | 894 | negative regulation of cellular metabolic process |
| GO:0016568 | 0,000787 | 1,940848 | 18,44822 | 33 | 320 | chromatin modification |
| GO:2000725 | 0,000809 | 9,161677 | 0,807109 | 5 | 14 | regulation of cardiac muscle cell differentiation |
| GO:0010605 | 0,00081 | 1,543081 | 53,38453 | 76 | 926 | negative regulation of macromolecule metabolic process |
| GO:0016202 | 0,000823 | 2,950705 | 5,361513 | 14 | 93 | regulation of striated muscle tissue development |
| GO:0060541 | 0,00085 | 2,526794 | 7,898143 | 18 | 137 | respiratory system development |
| GO:0000904 | 0,000878 | 1,807382 | 23,92503 | 40 | 415 | cell morphogenesis involved in differentiation |
| GO:1901861 | 0,000917 | 2,913465 | 5,419164 | 14 | 94 | regulation of muscle tissue development |
| GO:0008284 | 0,00092 | 1,802347 | 23,98268 | 40 | 416 | positive regulation of cell proliferation |
| GO:0001558 | 0,000938 | 2,312792 | 9,973567 | 21 | 173 | regulation of cell growth |
| GO:0021987 | 0,000961 | 3,445702 | 3,689643 | 11 | 64 | cerebral cortex development |

**Supplementary Table V B. GeneOntology of miR-141/200a targets, Molecular Function**

| GOMFID | Pvalue | OddsRatio | ExpCount | Count | Size | Term | |
| --- | --- | --- | --- | --- | --- | --- | --- |
| GO:0003700 | 1,85E-05 | 1,93220089 | 33,30471 | 58 | 583 | sequence-specific DNA binding transcription factor activity | |
| GO:0001071 | 1,94E-05 | 1,92827633 | 33,36184 | 58 | 584 | nucleic acid binding transcription factor activity | |
| GO:0003712 | 0,000178 | 2,29477612 | 13,48184 | 28 | 236 | transcription cofactor activity | |
| GO:0043565 | 0,000208 | 1,87128185 | 26,84943 | 46 | 470 | sequence-specific DNA binding | |
| GO:0000989 | 0,000228 | 2,18772305 | 15,08138 | 30 | 264 | transcription factor binding transcription factor activity | |
| GO:0008134 | 0,000362 | 2,01816355 | 18,39471 | 34 | 322 | transcription factor binding | |
| GO:0000988 | 0,000434 | 2,09542949 | 15,65264 | 30 | 274 | protein binding transcription factor activity | |
| GO:0005057 | 0,000434 | 3,84537894 | 3,37046 | 11 | 59 | receptor signaling protein activity | |
| GO:0043566 | 0,000569 | 2,54803414 | 8,283333 | 19 | 145 | structure-specific DNA binding | |
| GO:0004672 | 0,000632 | 1,87431643 | 21,99368 | 38 | 385 | protein kinase activity | |
| GO:0003690 | 0,000752 | 2,98073748 | 5,312759 | 14 | 93 | double-stranded DNA binding |  |

**Supplementary Table V C. GeneOntology of miR-141/200a targets,** Cellular Component

| GOCCID | Pvalue | OddsRatio | ExpCount | Count | Size | Term |
| --- | --- | --- | --- | --- | --- | --- |
| GO:0031519 | 0,000639 | 4,955422 | 1,998317 | 8 | 35 | PcG protein complex |

**Supplementary Table VI A. GeneOntology of miR-200b/200c/429/548a predicted targets, Biological Process**

| GOBPID | Pvalue | OddsRatio | ExpCount | Count | Size | Term |
| --- | --- | --- | --- | --- | --- | --- |
| GO:0006357 | 6,17E-06 | 1,647316 | 77,77031 | 115 | 915 | regulation of transcription from RNA polymerase II promoter |
| GO:2000113 | 1,48E-05 | 1,728425 | 55,16167 | 86 | 649 | negative regulation of cellular macromolecule biosynthetic process |
| GO:0006366 | 1,54E-05 | 1,610263 | 78,45027 | 114 | 923 | transcription from RNA polymerase II promoter |
| GO:0010558 | 2,08E-05 | 1,699119 | 57,28654 | 88 | 674 | negative regulation of macromolecule biosynthetic process |
| GO:0051253 | 2,2E-05 | 1,734385 | 51,67688 | 81 | 608 | negative regulation of RNA metabolic process |
| GO:0045934 | 2,59E-05 | 1,70249 | 55,16167 | 85 | 649 | negative regulation of nucleobase-containing compound metabolic process |
| GO:0051172 | 3,87E-05 | 1,680042 | 55,75664 | 85 | 656 | negative regulation of nitrogen compound metabolic process |
| GO:0031327 | 4,55E-05 | 1,656353 | 58,47647 | 88 | 688 | negative regulation of cellular biosynthetic process |
| GO:0009890 | 5,14E-05 | 1,645081 | 59,49641 | 89 | 700 | negative regulation of biosynthetic process |
| GO:0000122 | 5,29E-05 | 1,855617 | 34,59291 | 58 | 407 | negative regulation of transcription from RNA polymerase II promoter |
| GO:0006468 | 5,31E-05 | 1,606457 | 67,06095 | 98 | 789 | protein phosphorylation |
| GO:0045892 | 5,72E-05 | 1,700905 | 49,807 | 77 | 586 | negative regulation of transcription, DNA-dependent |
| GO:0031324 | 6,88E-05 | 1,560339 | 75,98542 | 108 | 894 | negative regulation of cellular metabolic process |
| GO:2000479 | 7,95E-05 | 27,08839 | 0,594964 | 5 | 7 | regulation of cAMP-dependent protein kinase activity |
| GO:0010628 | 0,000111 | 1,565664 | 69,18583 | 99 | 814 | positive regulation of gene expression |
| GO:0010608 | 0,000161 | 2,092483 | 19,20884 | 36 | 226 | posttranscriptional regulation of gene expression |
| GO:0045944 | 0,000169 | 1,662064 | 47,34214 | 72 | 557 | positive regulation of transcription from RNA polymerase II promoter |
| GO:0010629 | 0,000185 | 1,604346 | 55,75664 | 82 | 656 | negative regulation of gene expression |
| GO:0051254 | 0,000203 | 1,554571 | 65,19107 | 93 | 767 | positive regulation of RNA metabolic process |
| GO:2000480 | 0,000241 | 43,28841 | 0,424974 | 4 | 5 | negative regulation of cAMP-dependent protein kinase activity |
| GO:0021872 | 0,00027 | 3,962348 | 3,824769 | 12 | 45 | forebrain generation of neurons |
| GO:0051271 | 0,000271 | 2,703806 | 8,584482 | 20 | 101 | negative regulation of cellular component movement |
| GO:0048666 | 0,000319 | 1,643144 | 45,04728 | 68 | 530 | neuron development |
| GO:0010557 | 0,000344 | 1,510277 | 70,46075 | 98 | 829 | positive regulation of macromolecule biosynthetic process |
| GO:0045893 | 0,000371 | 1,53222 | 63,74615 | 90 | 750 | positive regulation of transcription, DNA-dependent |
| GO:0009892 | 0,000404 | 1,465387 | 82,955 | 112 | 976 | negative regulation of metabolic process |
| GO:0010605 | 0,000428 | 1,474534 | 78,70525 | 107 | 926 | negative regulation of macromolecule metabolic process |
| GO:2000146 | 0,000442 | 2,664744 | 8,244503 | 19 | 97 | negative regulation of cell motility |
| GO:0031175 | 0,000443 | 1,672987 | 39,01265 | 60 | 459 | neuron projection development |
| GO:0040013 | 0,000477 | 2,499535 | 9,604421 | 21 | 113 | negative regulation of locomotion |
| GO:0051246 | 0,000579 | 1,474497 | 74,11553 | 101 | 872 | regulation of protein metabolic process |
| GO:0043524 | 0,000589 | 2,771902 | 7,139569 | 17 | 84 | negative regulation of neuron apoptotic process |
| GO:0016358 | 0,000608 | 2,445737 | 9,77441 | 21 | 115 | dendrite development |
| GO:0002089 | 0,000632 | 6,329838 | 1,614903 | 7 | 19 | lens morphogenesis in camera-type eye |
| GO:0021602 | 0,000632 | 6,329838 | 1,614903 | 7 | 19 | cranial nerve morphogenesis |
| GO:0060235 | 0,000676 | 21,64151 | 0,509969 | 4 | 6 | lens induction in camera-type eye |
| GO:0030336 | 0,00072 | 2,622857 | 7,904523 | 18 | 93 | negative regulation of cell migration |
| GO:0048593 | 0,000762 | 2,922244 | 6,034636 | 15 | 71 | camera-type eye morphogenesis |
| GO:1901215 | 0,000821 | 2,588021 | 7,989518 | 18 | 94 | negative regulation of neuron death |
| GO:0031328 | 0,000896 | 1,451553 | 75,05047 | 101 | 883 | positive regulation of cellular biosynthetic process |
| GO:0032879 | 0,000899 | 1,426424 | 84,73989 | 112 | 997 | regulation of localization |
| GO:0045935 | 0,000936 | 1,459546 | 71,65068 | 97 | 843 | positive regulation of nucleobase-containing compound metabolic process |
| GO:0045844 | 0,000947 | 7,227027 | 1,274923 | 6 | 15 | positive regulation of striated muscle tissue development |
| GO:0048636 | 0,000947 | 7,227027 | 1,274923 | 6 | 15 | positive regulation of muscle organ development |

**Supplementary Table VI B. GeneOntology of miR-200b/200c/429/548a predicted targets, Molecular Function**

| GOMFID | Pvalue | OddsRatio | ExpCount | Count | Size | Term |
| --- | --- | --- | --- | --- | --- | --- |
| GO:0003690 | 8,76E-06 | 3,368703 | 7,985172 | 22 | 93 | double-stranded DNA binding |
| GO:0030971 | 2,03E-05 | 7,180461 | 2,146552 | 10 | 25 | receptor tyrosine kinase binding |
| GO:0004674 | 7,97E-05 | 1,991574 | 25,15759 | 45 | 293 | protein serine/threonine kinase activity |
| GO:0019899 | 0,000195 | 1,533918 | 71,09379 | 100 | 828 | enzyme binding |
| GO:0043566 | 0,000228 | 2,373967 | 12,45 | 26 | 145 | structure-specific DNA binding |
| GO:0005083 | 0,000506 | 2,069181 | 16,65724 | 31 | 194 | small GTPase regulator activity |
| GO:0030695 | 0,000534 | 1,84322 | 24,98586 | 42 | 291 | GTPase regulator activity |
| GO:0005283 | 0,000703 | 21,40242 | 0,515172 | 4 | 6 | sodium:amino acid symporter activity |
| GO:0060589 | 0,000934 | 1,783989 | 25,67276 | 42 | 299 | nucleoside-triphosphatase regulator activity |
| GO:0005158 | 0,000958 | 5,777547 | 1,717241 | 7 | 20 | insulin receptor binding |

**Supplementary Table VI C. GeneOntology of miR-200b/200c/429/548a predicted targets, Cellular Component**

| GOCCID | Pvalue | OddsRatio | ExpCount | Count | Size | Term |
| --- | --- | --- | --- | --- | --- | --- |
| GO:0031981 | 0,000658 | 1,51283 | 61,40561 | 86 | 727 | nuclear lumen |

**Supplementary Table VII A. GeneOntology of predicted targets with** two miR seeds. Biological Process

| GOBPID | Pvalue | OddsRatio | ExpCount | Count | Size | Term |
| --- | --- | --- | --- | --- | --- | --- |
| GO:0045595 | 4,82E-06 | 2,192744 | 25,41962 | 49 | 772 | regulation of cell differentiation |
| GO:0048699 | 4,88E-06 | 2,209029 | 24,69523 | 48 | 750 | generation of neurons |
| GO:0022008 | 4,91E-06 | 2,174328 | 26,17694 | 50 | 795 | neurogenesis |
| GO:0030182 | 1,13E-05 | 2,202245 | 22,52205 | 44 | 684 | neuron differentiation |
| GO:0048666 | 5,79E-05 | 2,225046 | 17,45129 | 35 | 530 | neuron development |
| GO:2000026 | 7,74E-05 | 1,940594 | 28,21841 | 49 | 857 | regulation of multicellular organismal development |
| GO:0006928 | 8,17E-05 | 1,989754 | 25,18913 | 45 | 765 | cellular component movement |
| GO:0000122 | 0,000168 | 2,295333 | 13,40128 | 28 | 407 | negative regulation of transcription from RNA polymerase II promoter |
| GO:0010628 | 0,000174 | 1,902864 | 26,80255 | 46 | 814 | positive regulation of gene expression |
| GO:0051254 | 0,000176 | 1,928811 | 25,25498 | 44 | 767 | positive regulation of RNA metabolic process |
| GO:0045893 | 0,000213 | 1,923753 | 24,69523 | 43 | 750 | positive regulation of transcription, DNA-dependent |
| GO:0051960 | 0,000215 | 2,257889 | 13,59884 | 28 | 413 | regulation of nervous system development |
| GO:0045664 | 0,000232 | 2,461884 | 10,24029 | 23 | 311 | regulation of neuron differentiation |
| GO:0031175 | 0,000242 | 2,175932 | 15,11348 | 30 | 459 | neuron projection development |
| GO:0010557 | 0,000265 | 1,862784 | 27,29646 | 46 | 829 | positive regulation of macromolecule biosynthetic process |
| GO:0046777 | 0,000269 | 3,459891 | 4,181725 | 13 | 127 | protein autophosphorylation |
| GO:0021559 | 0,000336 | 44,50699 | 0,164635 | 3 | 5 | trigeminal nerve development |
| GO:2000113 | 0,00034 | 1,951774 | 21,3696 | 38 | 649 | negative regulation of cellular macromolecule biosynthetic process |
| GO:0006357 | 0,000378 | 1,79695 | 30,12818 | 49 | 915 | regulation of transcription from RNA polymerase II promoter |
| GO:0050767 | 0,000422 | 2,262452 | 12,05127 | 25 | 366 | regulation of neurogenesis |
| GO:0031327 | 0,00054 | 1,884259 | 22,65375 | 39 | 688 | negative regulation of cellular biosynthetic process |
| GO:0007420 | 0,000641 | 2,273421 | 10,99761 | 23 | 334 | brain development |
| GO:2000794 | 0,000656 | 29,66783 | 0,197562 | 3 | 6 | regulation of epithelial cell proliferation involved in lung morphogenesis |
| GO:0010558 | 0,000708 | 1,869102 | 22,19278 | 38 | 674 | negative regulation of macromolecule biosynthetic process |
| GO:0045935 | 0,000723 | 1,77724 | 27,75743 | 45 | 843 | positive regulation of nucleobase-containing compound metabolic process |
| GO:0010464 | 0,000747 | 6,405856 | 1,119517 | 6 | 34 | regulation of mesenchymal cell proliferation |
| GO:0009890 | 0,000755 | 1,847219 | 23,04888 | 39 | 700 | negative regulation of biosynthetic process |
| GO:0051253 | 0,000805 | 1,903402 | 20,0196 | 35 | 608 | negative regulation of RNA metabolic process |
| GO:0045892 | 0,000836 | 1,916908 | 19,2952 | 34 | 586 | negative regulation of transcription, DNA-dependent |
| GO:0006366 | 0,000841 | 1,732894 | 30,39159 | 48 | 923 | transcription from RNA polymerase II promoter |
| GO:0007417 | 0,000858 | 2,078287 | 14,09274 | 27 | 428 | central nervous system development |
| GO:0060284 | 0,000913 | 2,040338 | 14,88299 | 28 | 452 | regulation of cell development |
| GO:0051173 | 0,000996 | 1,745796 | 28,18548 | 45 | 856 | positive regulation of nitrogen compound metabolic process |

**Supplementary Table VII B. GeneOntology of predicted targets with two miR seeds. Molecular Function**

| GOMFID | Pvalue | OddsRatio | ExpCount | Count | Size | Term |
| --- | --- | --- | --- | --- | --- | --- |
| GO:0004672 | 0,00049 | 2,200032 | 12,87759 | 26 | 385 | protein kinase activity |
| GO:0004674 | 0,000803 | 2,326757 | 9,800345 | 21 | 293 | protein serine/threonine kinase activity |
| GO:0016301 | 0,000922 | 1,965201 | 17,12552 | 31 | 512 | Kinase activity |

**Supplementary Table VII C. GeneOntology of predicted targets with two miR seeds.**  Cellular Components

| GOCCID | Pvalue | OddsRatio | ExpCount | Count | Size | Term |
| --- | --- | --- | --- | --- | --- | --- |
| GO:0031519 | 0,000794 | 6,308144 | 1,130679 | 6 | 35 | PcG protein complex |

**Supplementary Table VIII A. GeneOntology of predicted targets with three miR seeds.**  Biological Process

| GOBPID | Pvalue | OddsRatio | ExpCount | Count | Size | Term |
| --- | --- | --- | --- | --- | --- | --- |
| GO:0045892 | 7,44E-06 | 6,7938 | 2,270024 | 11 | 586 | negative regulation of transcription, DNA-dependent |
| GO:0051253 | 1,06E-05 | 6,525817 | 2,355247 | 11 | 608 | negative regulation of RNA metabolic process |
| GO:0000122 | 1,69E-05 | 7,548241 | 1,576621 | 9 | 407 | negative regulation of transcription from RNA polymerase II promoter |
| GO:0045934 | 1,97E-05 | 6,075712 | 2,514071 | 11 | 649 | negative regulation of nucleobase-containing compound metabolic process |
| GO:2000113 | 1,97E-05 | 6,075712 | 2,514071 | 11 | 649 | negative regulation of cellular macromolecule biosynthetic process |
| GO:0010629 | 2,18E-05 | 6,004584 | 2,541187 | 11 | 656 | negative regulation of gene expression |
| GO:0051172 | 2,18E-05 | 6,004584 | 2,541187 | 11 | 656 | negative regulation of nitrogen compound metabolic process |
| GO:0010558 | 2,81E-05 | 5,828579 | 2,610915 | 11 | 674 | negative regulation of macromolecule biosynthetic process |
| GO:0031327 | 3,4E-05 | 5,698157 | 2,665148 | 11 | 688 | negative regulation of cellular biosynthetic process |
| GO:0009890 | 3,99E-05 | 5,590585 | 2,711633 | 11 | 700 | negative regulation of biosynthetic process |
| GO:0006357 | 9,53E-05 | 4,735729 | 3,544491 | 12 | 915 | regulation of transcription from RNA polymerase II promoter |
| GO:0006366 | 0,000104 | 4,689352 | 3,575481 | 12 | 923 | transcription from RNA polymerase II promoter |
| GO:0000904 | 0,000143 | 6,302022 | 1,607611 | 8 | 415 | cell morphogenesis involved in differentiation |
| GO:0031324 | 0,000363 | 4,257226 | 3,463142 | 11 | 894 | negative regulation of cellular metabolic process |
| GO:0010605 | 0,000493 | 4,091613 | 3,587103 | 11 | 926 | negative regulation of macromolecule metabolic process |
| GO:0007422 | 0,000674 | 20,04839 | 0,174319 | 3 | 45 | peripheral nervous system development |
| GO:0009892 | 0,000774 | 3,854832 | 3,780791 | 11 | 976 | negative regulation of metabolic process |
| GO:0043506 | 0,000974 | 17,53024 | 0,197562 | 3 | 51 | regulation of JUN kinase activity |
| GO:0048667 | 0,00099 | 6,051601 | 1,181497 | 6 | 305 | cell morphogenesis involved in neuron differentiation |

**Supplementary Table VIII B. GeneOntology of predicted targets with three miR seeds.**  Cellular Components

| GOCCID | Pvalue | OddsRatio | ExpCount | Count | Size | Term |
| --- | --- | --- | --- | --- | --- | --- |
| GO:0016605 | 0,000799 | 18,8267 | 0,18452 | 3 | 47 | PML body |
