# Supplementary figures and images for: The Dlx5 and Foxg1 transcription factors, linked via miRNA-9 and -200, are required for the development of the olfactory and GnRH system
Source: Mol Cell Neurosci. 2015 Sep;68:103–19. doi: 10.1016/j.mcn.2015.04.007 (PMC4604252; doi:10.1016/j.mcn.2015.04.007)

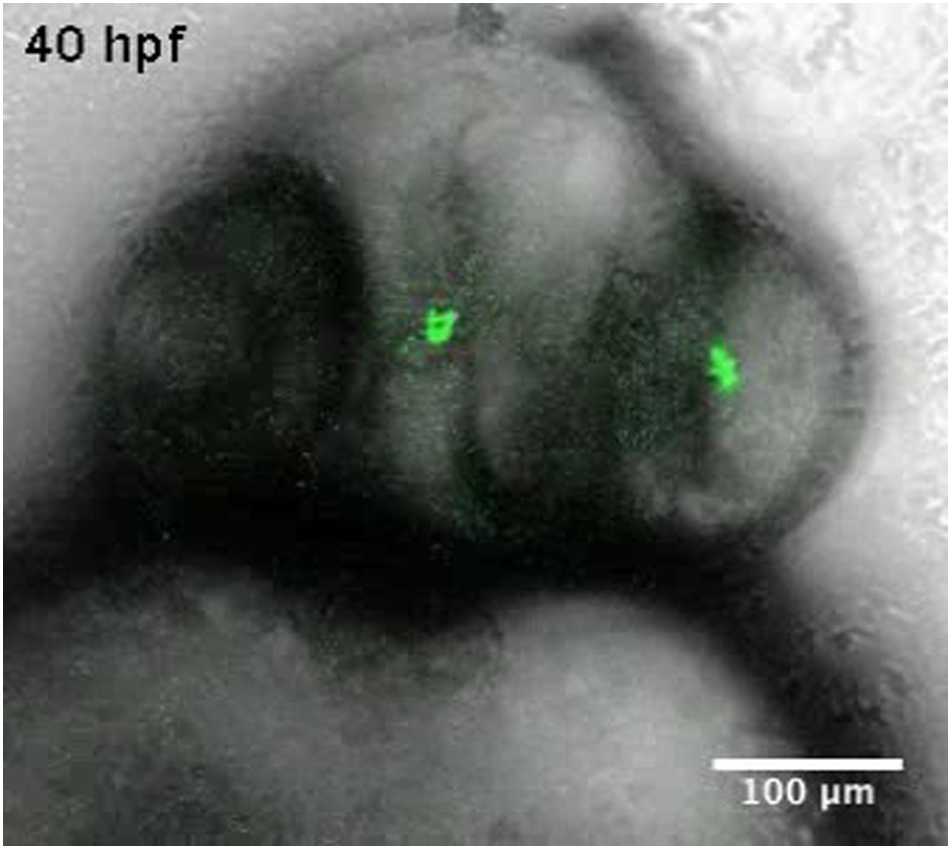

Supplement: Supplementary file 3 — Supplementary video 1. [file mmc3.jpg]

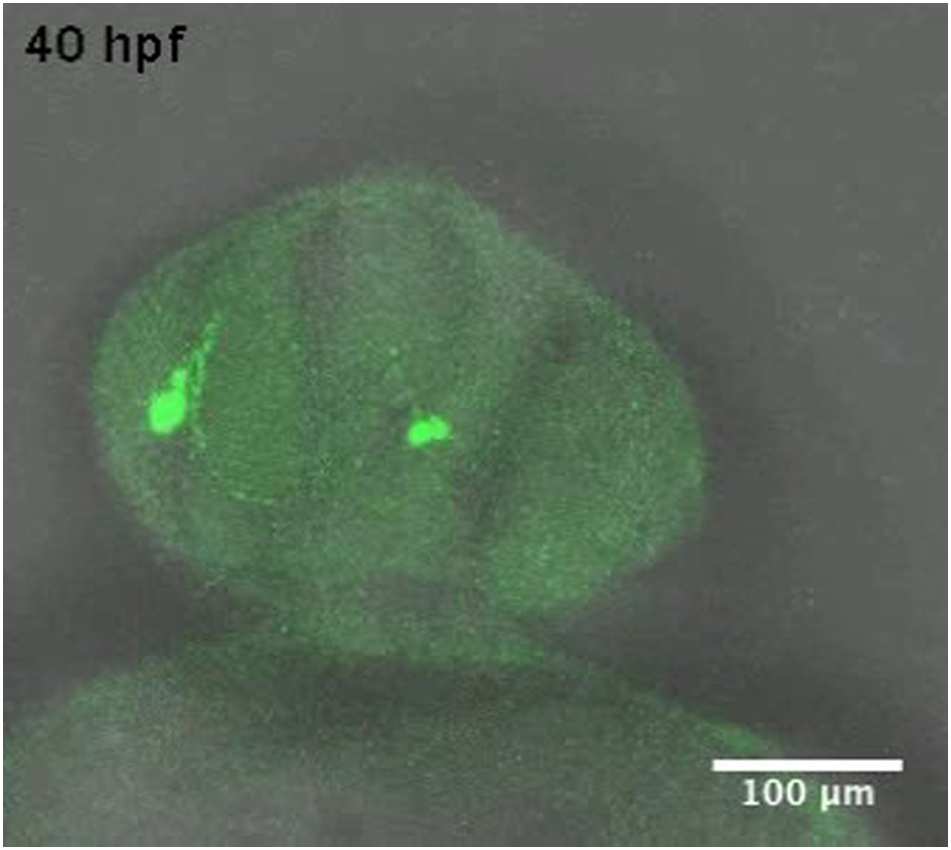

Supplement: Supplementary file 4 — Supplementary video 2. [file mmc4.jpg]
